# Knocking out USP7 attenuates cardiac fibrosis and endothelial-to-mesenchymal transition by destabilizing SMAD3 in mice with heart failure with preserved ejection fraction

**DOI:** 10.7150/thno.97767

**Published:** 2024-09-09

**Authors:** Shuai Yuan, Zimu Wang, Shun Yao, Yanyan Wang, Zhonglei Xie, Jingfeng Wang, Xueting Yu, Yu Song, Xiaotong Cui, Jingmin Zhou, Junbo Ge

**Affiliations:** Department of Cardiology, Shanghai Institute of Cardiovascular Diseases, Zhongshan Hospital, Fudan University, Shanghai, China.

**Keywords:** USP7, Deubiquitination enzyme, Heart failure with preserved ejection fraction, Endothelial-to-mesenchymal transition, Cardiac fibrosis

## Abstract

**Background**: Heart failure with preserved ejection fraction (HFpEF) is a predominant type of heart failure. Exploring new pathogenesis and identifying potential novel therapeutic targets for HFpEF is of paramount importance.

**Methods**: HFpEF mouse model was established by the "Multiple-hit" strategy, in that 18- to 22-month-old female C57B6/J mice fed with a high-fat diet were further challenged with chronic infusion of Angiotensin II. RNA sequencing analysis showed that USP7 was significantly increased in the heart of HFpEF mice. Liquid chromatography coupled with tandem mass spectrometry (LC-MS/MS) analysis, in conjunction with co-immunoprecipitation (Co-IP) techniques, identified expression of SMAD3, the key molecule of endothelial-to-mesenchymal transition (EndMT), was also significantly elevated. USP7 endothelium-specific knockout mice was generated to investigate the involvement of USP7 in HFpEF. The biological significance of the interaction between USP7 and SMAD3 was further explored.

**Results**: USP7 promotes EndMT and cardiac fibrosis by binding to SMAD3 directly via its UBL (Ubiquitin-like) domain and cysteine at position 223 of USP7, leading SMAD3 deubiquitination to maintain the stability of SMAD3 by removing the K63 ubiquitin chain and preventing the degradation of SMAD3 by proteasomal process. USP7 also promotes SMAD3 phosphorylation and nuclear translocation, thereby aggravating EndMT and cardiac fibrosis. Endothelium-specific USP7 knockout led to improvement of HFpEF phenotypes and reduction of cardiac fibrosis. Overexpression of SMAD3 in endothelium-specific knockout HFpEF mice reversed the protective effects of USP7 knockout in this HFpEF mouse model.

**Conclusion:** Our results indicated that USP7 is one of the key pathogenic molecules of HFpEF, and knocking out USP7 could attenuate HFpEF injury by promoting the degradation of SMAD3. USP7 and SMAD3 inhibition might be potential therapeutic options for HFpEF.

## Introduction

Heart failure (HF) is a clinical syndrome causing significant morbidity, mortality, and healthcare expenditure. Heart failure with preserved ejection fraction (HFpEF) is a predominant type of heart failure, representing approximately 50% of all HF cases^1^. Given the aging population and the ongoing epidemics of metabolic disorders and hypertension, the prevalence of HFpEF is expected to rising continuously in the future^2^, ^3^. However, HFpEF has shown poor response to the standard treatment approach used for heart failure with reduced ejection fraction (HFrEF). Major clinical trials conducted have not yielded positive results on primary outcomes until the era of angiotensin receptor-neprilysin inhibitor (ARNI) sacubitril/valsartan and the sodium-glucose cotransporter 2 inhibitor (SGLT2i) empagliflozin^4^-^6^. Exploring the pathogenesis and identifying potential therapeutic targets for HFpEF is thus of paramount importance to develop novel therapeutic targets. There is growing recognition that cardiac fibrosis plays a significant role in the etiology of all types of HF, particularly in the pathophysiology of HFpEF^7^, ^8^. Among the multiple factors contributing to the development of HFpEF, fibrosis serves as a major pathogenic factor irrespective of the underlying etiology. It has been observed that the extracellular fibrotic burden exhibits a stronger correlation with diastolic dysfunction and is also associated with increased hospitalization and mortality in HFpEF^9^, ^10^. Options to attenuate fibrosis thus draw significant attention on alleviating HFpEF phenotypes. However, in contrast to cardiac fibrosis observed in HFrEF, characterized by the replacement of cardiomyocyte loss with extracellular matrix (ECM) proteins to preserve the structural integrity of the myocardium, cardiac fibrosis in HFpEF is considered to be reactive fibrosis occurring in the context of systemic inflammation and metabolic abnormalities in the setting of non-significant cardiomyocyte death^11^. In this reactive fibrosis process, a series of reactions resulted from endothelial inflammation, such as impaired nitric oxide (NO) utilization, and increased adhesion of inflammatory cells, which serve as significant driving factors for enhanced fibrosis and ventricular remodeling in HFpEF^12^. Under conditions of systemic inflammation, dysfunctional endothelial cells (ECs) have been observed to undergo a phenotypic transformation into a mesenchymal-like state, commonly referred to as endothelial-mesenchymal transition (EndMT)^13^, ^14^. Previous studies have demonstrated that EndMT could contribute to the process of cardiac fibrosis^15^-^17^. While myofibroblasts are traditionally recognized as the primary contributors to fibrosis, there is an increasing recognition of the crucial role played by EndMT in myocardial fibrosis. It is known that EndMT may function as an intermediate process linking endothelial inflammation and ventricular remodeling in HFpEF^18^. Thus, targeting EndMT might emerge as a novel therapeutic strategy for managing HFpEF.

Ubiquitination, as a crucial posttranslational modification, plays a significant role in various cellular processes including cell signal transduction, cell fate determination, inflammatory responses, and other essential biological activities^19^, ^20^. Ubiquitination is a reversible process that can be counter-regulated by deubiquitinating enzymes (DUBs). DUBs are essential for maintaining cellular signaling networks and are involved in various aspects of pathophysiology by precisely controlling protein function, localization, and degradation. Approximately 100 deubiquitinating enzymes (DUBs) have been identified, and they play a significant role in regulating intracellular signal transduction. These DUBs have been found to be closely associated with various cardiovascular diseases^21^-^23^. However, the exact involvement of DUBs in the pathogenesis of HFpEF is not fully understood.

In our experiment, we examined the expression of DUBs in HFpEF mice induced by a combined set of risk factors, including age, obesity, and hypertension, referred to as the "Multiple-hit" Strategy^24^. We identified significantly upregulated level of a DUB, USP7(ubiquitin-specific protease 7), also known as herpes virus-associated ubiquitin-specific protease (HAUSP), in heart tissues of HFpEF mice. Furthermore, our findings revealed that the observed increase in USP7 expression was predominantly localized within endothelial cells in HFpEF mice. To examine the specific role of USP7 in endothelial cells, we generated endothelial cell-specific USP7 knockout mice. Our results provided evidence that the deficiency of USP7 in endothelial cells improved diastolic dysfunction, reduced BNP, fibrosis and EndMT in HFpEF model. Detailed mechanistic studies showed that USP7 deficiency blocked its interaction with SMAD3, leading to enhanced degradation and inactivation of SMAD3. SMAD3 overexpression reversed the protective effects of USP7 deficiency in this HFpEF model. Our results thus hint that targeting USP and/or SMAD3 might serve as promising therapeutic options for the treatment of HFpEF.

## Methods

All data, methods, and study materials will be made available to other researchers for the purposes of reproducing our results or replicating the procedures. Detailed methods are provided in the Supplemental Material.

### Animal studies

WT mice (C57BL/6J) were purchased from Beijing Vital River Laboratory Animal Technology Co., Ltd (Beijing, China). *USP7*^flox/flox^ mice and Cdh5-Cre^ERT^ mice on C57BL/6J background, aged 8-10 months, were purchased from Cyagen (Suzhou, China). Endothelial-specific conditional USP7 deficiency mice (*USP7*^flox/flox^/Cdh5-Cre^ERT^) were generated by crossing *USP7*^flox/flox^ mice with Cdh5-Cre^ERT^ mice and intraperitoneally injected with tamoxifen (30 mg/kg) daily for 5 days. All animal experiments complied with the Guide for the Care and Use of Laboratory Animals published by the US National Institutes of Health (publication No. 85-23, revised 1996) and permitted by the Animal Care and Use Committee of Zhongshan Hospital, Fudan University.

### Statistical analysis

Data were reported as Mean ± SEM. For n ≥ 6 data, the Shapiro-Wilk normality test was conducted to assess the normality of the data. Fisher's exact test was utilized to compare categorical variables. For data with a normal distribution, the unmatched two-tailed Student's t-test was employed to determine whether the difference between the two groups was statistically significant. For multi-group comparison, one-way or two-way ANOVA with Tukey's multiple comparison test or Šidák test was utilized. For datasets with n < 6 or non-normal distribution, the non-parametric unpaired Mann-Whitney test was used to assess the statistical significance of the difference between the two groups. A statistically significant difference was obtained at P < 0.05. Data were analyzed by GraphPad Prism software (version 9.4.1, CA, USA) and R (Version 4.2.3).

## Results

### Endothelial USP7 expression is upregulated in HFpEF mice generated by the Mutiple-hit strategy

**Figure 1:**
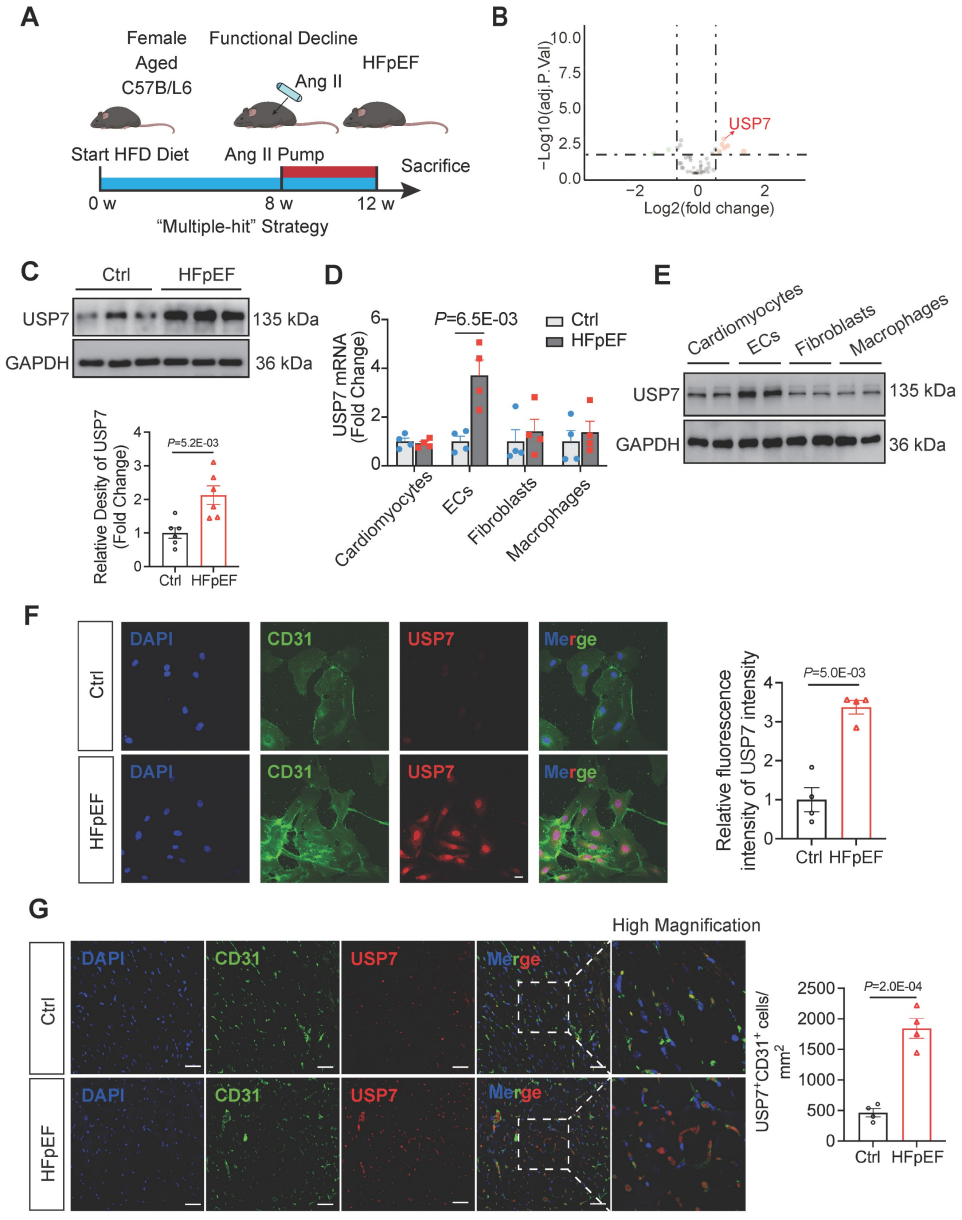
**Endothelial USP7 expression was upregulated in HFpEF mice generated by a “Multiple-hit” strategy. A**, A schematic representation of the “Multiple-hit” strategy. **B**, RNA transcriptome sequencing was used to reveal the expression profile of DUBs (deubiquitinating enzymes) in HFpEF mice. RNA transcriptome sequencing was performed on control (n = 4) and HFpEF (n = 4) mice heart samples, respectively. We use log2 of the fold change as the source of data for the x axis and -log10 of the P as the source of data for the y axis. Fold change ˃ 1.5× and P < 0.05 indicate statistically significant differences. Red and green points represent the upregulated DUBs and the downregulated DUBs compared with control group. Red arrow labeled represents the exact point of USP7; Although, black points represent the DUBs with no statistical difference compared with the control group. **C**, Representative western blotting for USP7 in normal heart tissue and HFpEF heart tissue in HFpEF mice and densitometric quantification of USP7, n = 6. **D**, Real-time qPCR analysis of the mRNA expression of USP7 in primary cardiomyocytes, primary ECs, cardiac fibroblast and macrophages isolated from HFpEF mice heart tissue. **E**, Representative western blot analysis for USP7 protein levels in primary cardiomyocytes, ECs, cardiac fibroblast and macrophages. GAPDH was used as loading control. **F**, Immunofluorescence staining and quantification of USP7 (red) and CD31 (green) in the isolated primary ECs from HFpEF mice. Scale bars, 20 μm. **G**, Immunofluorescence staining of USP7(red) and CD31(green) on cross sections of the heart tissues from mice under “Multiple-hit” strategy insult and mice under normal diet. Scale bars, 20 μm. **C-F**: Student t test; number of comparisons = 12(**C**), number of comparisons = 8(**D-F**); DAPI indicates 4'6-diamidino-2-phenylindole; HFD, high-fat diet; ECs, endothelial cells; HFpEF, heart failure with preserved ejection fraction; The protein level was standardized by GAPDH from each group was normalized to 1 value from the control group, which was set to 1.

To establish the HFpEF phenotypes, female mice (18 - 22 months old) were fed a high-fat diet for 12 weeks and infused with angiotensin II (Ang II) at a dosage of 1.25 mg/kg/day from the 8th week to the 12th week (**Figure 1A**). These HFpEF mice recapitulate human HFpEF by demonstrating hypertension, obesity, exercise intolerance, lung congestion, left ventricular (LV) hypertrophy, and hemodynamic evidence of diastolic dysfunction, featured by higher E/e', while LV ejection fraction (EF) remains preserved (**Figure S1-2**). Histopathologic data showed that hearts of HFpEF mice exhibited cardiac hypertrophy, pronounced collagen deposition, microvascular rarefaction and interstitial fibrosis (**Figure S3**). Recent years have witnessed an increasing body of evidence implicating DUBs in the pathogenesis of heart failure. Through transcriptome sequencing, we observed significantly differentiated expression of a large number of DUBs in the myocardial tissue of HFpEF mice (**Figure 1B, Figure S5, Table S1**). Additionally, mRNA expression of the DUBs was detected through transcriptome sequencing and results showed that the transcription of USP7 was significantly upregulated in HFpEF mice (**Figure 1B-C**). Immunofluorescence staining on the extracted cells from the hearts of HFpEF mice and subsequent western blotting experiments showed that the upregulation of USP7 was primarily localized in the endothelial cells (**Figure 1D through 1G, Figure S4**). Collectively, these results indicated the upregulation of endothelial USP7 in this HFpEF model.

### Endothelium-specific knockout of USP7 alleviates cardiac fibrosis by mitigating EndMT, thereby ameliorating the HFpEF phenotypes

**Figure 2:**
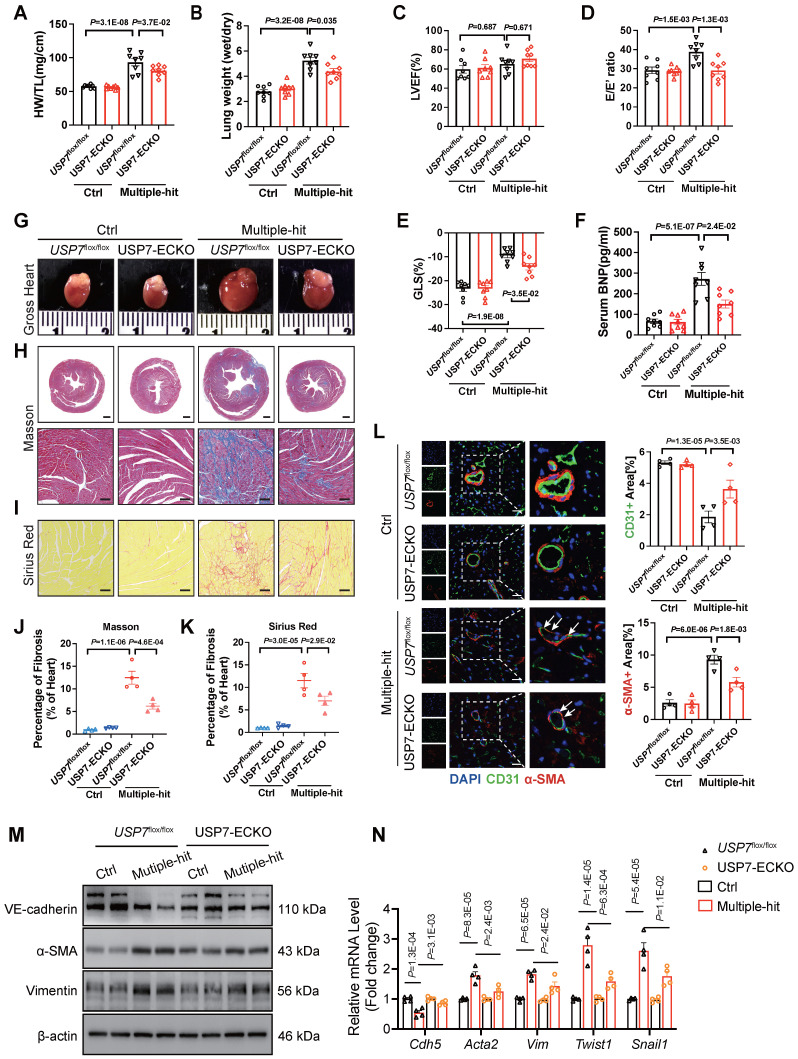
** EC-specific knockout of USP7 alleviates cardiac fibrosis by mitigating EndMT, thereby ameliorating the HFpEF phenotypes.** *USP7*^flox/flox^ and USP7-ECKO mice were subjected to normal diet and “Multiple-hit” strategy. **A**, Representative whole heart image from mice in each group. **B** and **C**, Masson (**B**) and Sirius Red (**C**) in sections of hearts. (Scale bar, 1 mm and 100 μm for Masson; 100 μm for Sirius Red staining). **D and E**, Quantification of fibrosis by assessing the Masson(**O**) and SR-positive(**P**) areas, n = 4. **F**, Heart weight (HW) normalized to tibia length (TL). **G**, Ratio between wet and dry lung weight. **H**, Percentage of left ventricular ejection fraction (LVEF). **I**, Ratio between mitral E wave and E' wave (E/E'). **J**, Percentage of global longitudinal strain (GLS). **K**, The serum levels of BNP (B-type natriuretic peptide) in four groups. **L**, Representative immunofluorescent staining images and quantification of microvascular endothelial cell CD31 (green) and fibrosis marker α-SMA (red) in the heart tissues (n = 4). Scale bar, 20 μm. **M**, Representative western blot analysis for endothelial cell marker (VE-cadherin), mesenchymal marker (α-SMA, Vimentin) from the heart tissues of four groups. **N**, Real-time qPCR analysis of endothelial cell marker (*Cdh5*), mesenchymal marker (*Acta2, Vim*) and transcription factors of EndMT (*Twist1, Snail1*) in heart tissues. n = 4. **D-L, N**: 1-way ANOVA followed by Tukey post-hoc tests; Data are shown as mean±SEM and adjusted P values were provided in case of multiple groups. The protein level was standardized by GAPDH and the mRNA level from each group was normalized to 1 value from the control group, which was set to 1.

USP7 knockout led to early embryonic lethality^25^. Endothelial-specific conditional USP7 deficiency mouse (*USP7*^flox/flox^/Cdh5-Cre^ERT^; USP7-ECKO) were generated by crossing *USP7*^flox/flox^ mice with Cdh5-Cre^ERT^ mice and intraperitoneally injected with tamoxifen (30 mg/kg) daily for 5 days (**Figure S6**). To examine the functional significance of endothelial USP7 activation in HFpEF mice, *USP7*^flox/flox^ and USP7-ECKO mice were stimulated with the “Multiple-hit” strategy. After “Multiple-hit” strategy, USP7-ECKO mice exhibited a lower heart weight-to-tibia length ratio (HW/TL), lower lung weight wet/dry ratio, and improved exercise tolerance compared to *USP7*^flox/flox^ mice (**Figure 2A and 2B, Figure S7B**). However, no significant difference in body weight and blood pressure were observed between 2 groups (**Figure S7A**). Serum BNP levels and TGFβ1 levels were found to be decreased in USP7-ECKO mice compared to *USP7*^flox/flox^ HFpEF mice (**Figure 2F, Figure S7J**). Echocardiography results demonstrated partial improvement in diastolic function in mice with USP7-ECKO mice, as reflected by E/e', -GLS and IVRT as compared to *USP7*^flox/flox^ HFpEF mice (**Figure 2C through 2E, Figure S7E**). Tissue section staining revealed that cardiac fibrosis and microvascular rarefaction were ameliorated, and EndMT was alleviated in mice with EC-specific knockout of USP7 (**Figure 2J through 2L, Figure S8**). Western blot assay and RT-qPCR further demonstrated that the EC-specific knockout of USP7 could alleviate the reduction in endothelial phenotype expression and the increase in interstitial phenotype expression induced by the “Multiple-hit” strategy (**Figure 2M and 2N, Figure S7K**).

### USP7 is involved in endothelial EndMT *in vitro*

**Figure 3:**
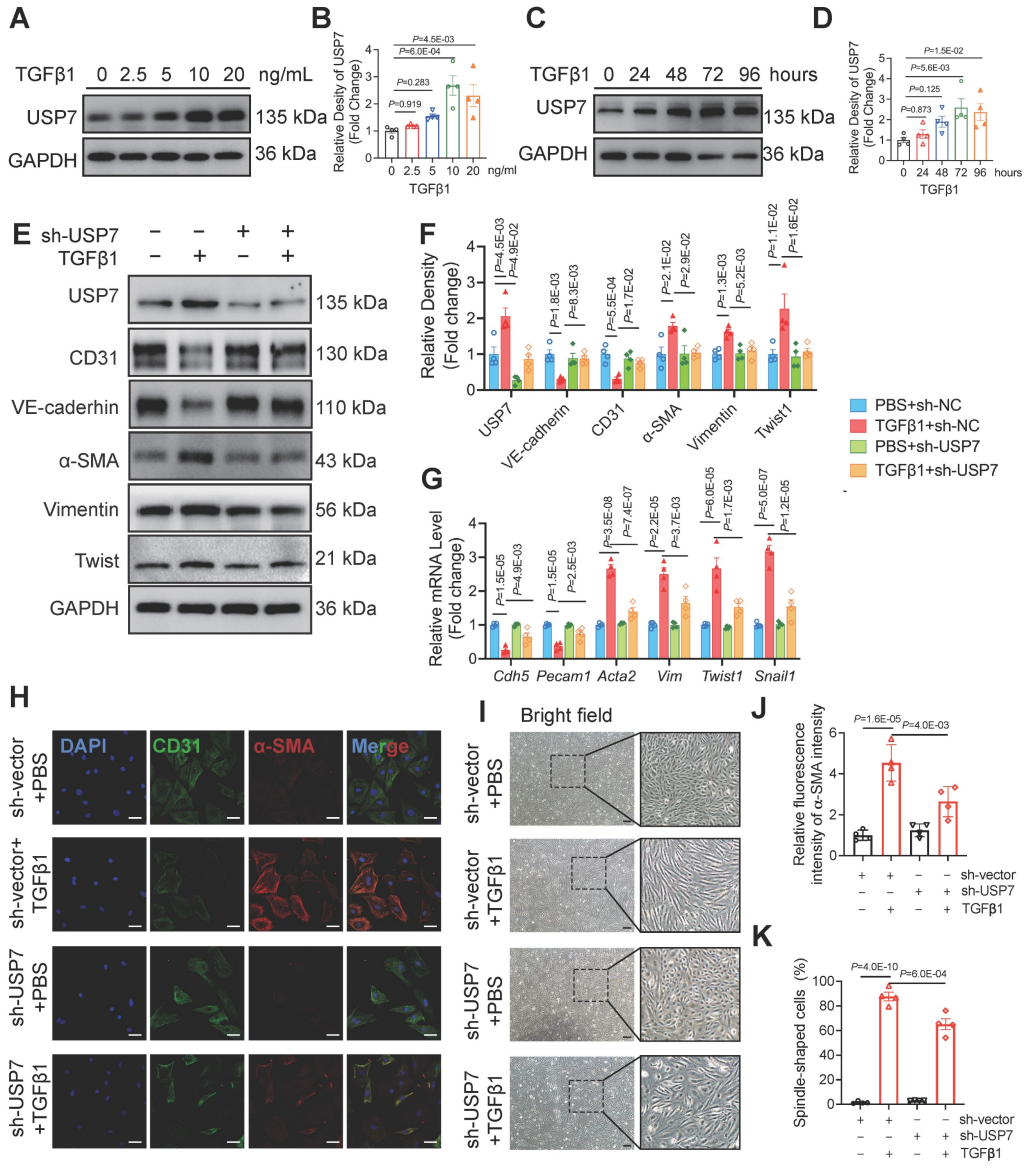
** USP7 is involved in endothelial EndMT *in vitro*. A** and **B**, Representative western blotting analysis (**A**) and densitometric quantification (**B**) of USP7 under different TGFβ1 stimulation in primary cardiac microvascular endothelial cells (CMECs). n =4. **C** and **D**, Representative western blotting analysis (**C**) and densitometric quantification (**D**) of USP7 under different treating time of TGFβ1 stimulation(10ng/ml) in primary cardiac microvascular endothelial cells (CMECs). n = 4. **E** and **F**, Representative western blotting analysis (**E**) and densitometric quantification (**F**) of endothelial cell marker (CD31, VE-cadherin), mesenchymal marker (α-SMA, Vimentin) and transcription factors of EndMT (Twist1) in primary cardiac microvascular endothelial cells (CMECs) transfected with short hairpin RNA lentiviral particles targeting USP7 (shUSP7) or control adenovirus (sh-Vector) under TGFβ1 stimulation (10ng/ml, 72h) or PBS control. n = 4. **G**, Expression analysis by RT-qPCR of endothelial marker (Cdh5 and Pecam1), mesenchymal marker (Acta2 and Vim) and transcription factors of EndMT (Twist1 and Snail1) in primary cardiac microvascular endothelial cells (CMECs) transfected with short hairpin RNA lentiviral particles targeting USP7 (shUSP7) or control adenovirus (sh-Vector) under TGFβ1 stimulation (10ng/ml, 72h) or PBS control. n = 4. **H** and **I**, Immunofluorescence staining of CD31 (green) and α-SMA (red) (**H**) and bright field image (**I**) in transfected CMECs (sh-Vector or sh-USP7) were either untreated or treated with TGFβ1 for 72 hours. Scale bars, 20 μm(**H**), 200 μm(**I**). **J** and **K**, Quantification of EndMT of CMECs by assessing the α-SMA intensity(**J**) of **H** and percentage of spindle-shaped cells(**K**) in **I,** n = 4. **B, D:** 2-way ANOVA followed by Dunnett's multiple comparisons tests; **F, G, J, K:** 1-way ANOVA followed by Tukey post-hoc tests; Data are shown as mean±SEM and adjusted P values were provided in case of multiple groups. The protein level was standardized by GAPDH, the mRNA level and α-SMA intensity from each group was normalized to 1 value from the control group, which was set to 1.

Based on the increased expression of USP7 in endothelial cells of HFpEF mice and the observed partial improvement in cardiac fibrosis and EndMT following EC-specific knockout of USP7 in this *in vivo* HFpEF model, we hypothesized that USP7 might alleviate HFpEF cardiac fibrosis by regulating the EndMT process in the setting of HFpEF. To prove this concept, we isolated primary cardiac microvascular endothelial cells (CMECs) from lactating rats. Results showed that USP7 expression was upregulated in the process of EndMT upon TGFβ1 stimulation in a time-dependent manner and the most significant increase in USP7 expression upon TGFβ1 stimulation was found at the concentration of 10 ng/ml, consistent with the observations in the animal model (**Figure 3A Through 3D**). Additionally, reducing the protein expression of USP7 through short hairpin RNA lentiviral particles targeting USP7 (shUSP7) transfection resulted in the amelioration of EndMT under TGFβ1 stimulation, characterized by an increase in the endothelial cell phenotype and a decrease in the interstitial phenotype (**Figure 3E through 3K, Figure S10**). These findings suggest that USP7 plays a crucial role in the regulation of EndMT in cardiac endothelial cells.

### USP7 directly interacts with SMAD3, with SMAD3 being one of the crucial substrates of USP7

**Figure 4:**
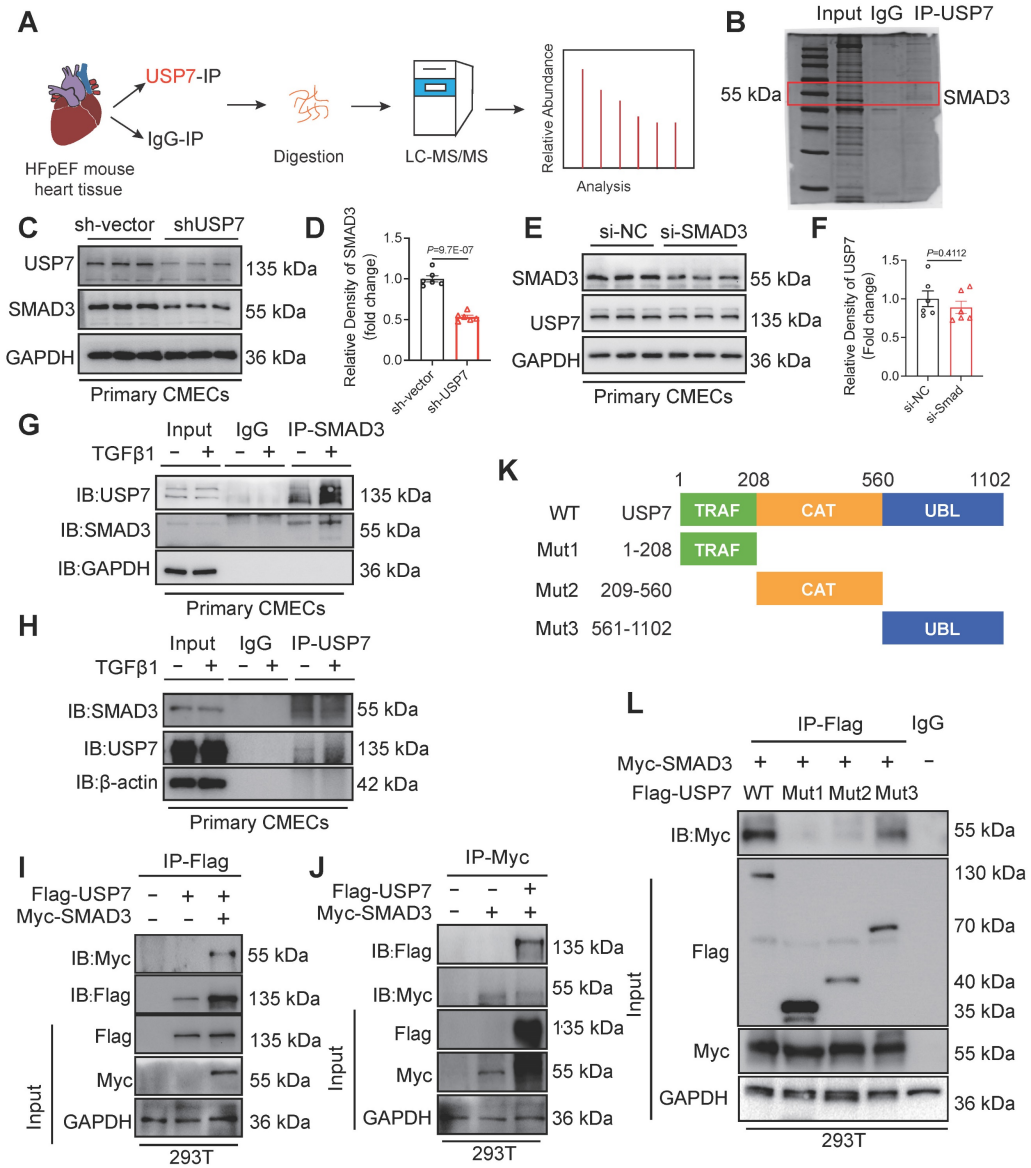
** USP7 directly interacts with SMAD3. A**, Schematic illustration of quantitative proteomic screen. **B**, Coomassie Blue staining of potential target proteins of USP7. **C** and **D**, CMECs were transfected with short hairpin RNA lentiviral particles targeting USP7 (shUSP7) for 24 h, while the control cells were transfected with control adenovirus (sh-Vector), Levels of USP7 and SMAD3 protein were measured by western blotting analysis (**C**) and densitometric quantification (**D**), n = 6. **E** and **F**, CMECs were transfected with SMAD3 siRNA for 24 h, while the control cells were transfected with negative control (NC) siRNA. Levels of SMAD3 and USP7 protein were measured by western blotting analysis (**E**) and densitometric quantification (**F**), n = 6. **G** and **H**, Coimmunoprecipitation of USP7 and SMAD3 in CMECs treated with or without TGFβ1 stimulation. Endogenous USP7 was immunoprecipitated by anti-USP7 antibody (**G**) and Endogenous SMAD3 was immunoprecipitated by anti-SMAD3 antibody (**H**). IgG, immunoglobulin G. n = 3. **I** and **J**, Coimmunoprecipitation of USP7 and SMAD3 in 293T cells co-transfected with Flag-USP7 and Myc-SMAD3 plasmids. Exogenous USP7 was immunoprecipitated by anti-Flag antibody(**I**) and exogenous SMAD3 was immunoprecipitated by anti-Myc antibody(**J**), n = 3. **K**, Schematic illustration of the USP7 domain deletion construct used in **L**. **L**, Coimmunoprecipitation of WT-USP7, Mut-USP7, and SMAD3 in 293T cells co-transfected with overexpression plasmids of Flag-WT-USP7, Flag-Mut-USP7 and Myc-SMAD3. Exogenous normal or mutated USP7 was immunoprecipitated by anti-Flag antibody. n = 3. **D, F**: Student t test; number of comparisons = 6; The protein level was standardized by GAPDH from each group was normalized to 1 value from the sh-Vector/si-NC group, which was set to 1.

It is known that DUBs could modulate biological activities by influencing the degradation or function of substrate proteins^26^. In order to identify the substrate proteins involved in EndMT and regulated by USP7, we extracted the proteins of cardiac tissue from HFpEF mice and conducted a screening of potential substrate proteins using co-immunoprecipitation (Co-IP) in conjunction with Liquid chromatography coupled with tandem mass spectrometry (LC-MS/MS) analysis. After excluding the peptides related to the light and heavy chains of the antibody, SMAD3, a key molecule involved in the regulation of EndMT, was found to be a potential substrate for USP7 (**Figure 4A and 4B**). As shown in **Figure 4C and 4D**, knockdown of USP7 expression resulted in a reduction in the expression of SMAD3, indicating a regulatory relationship in that USP7 could influence SMAD3 expression. However, the reverse scenario, where knocking down the expression of SMAD3 does not have a significant impact on USP7 expression, suggests that SMAD3 is the downstream signaling of USP7 (**Figure 4E and 4F**). Furthermore, TGFβ1 stimulation further enhanced the interaction between USP7 and SMAD3 in CMECs (**Figure 4G and 4H**). Subsequently, USP7 and SMAD3 plasmids were co-transfected into 293T cells and their interaction was confirmed by visualizing the co-localization of USP7 and SMAD3 (**Figure 4I and 4J**). USP7 consists of 3 domains: a TRAF (TNF receptor-associated factor) domain, a CAT (Cysteine-rich domain Associated with TRAF1) domain, and a UBL (Ubiquitin-like) domain^27^. To further elucidate the specific domain of USP7 that interacts with SMAD3, three USP7 mutants were generated. Results of co-transfection of SMAD3 and the respective mutated USP7 plasmids in 293T cells showed that the USP7 mutant containing amino acids 561-1102 retained the ability to bind to SMAD3, while mutants in other domains failed to interact with SMAD3 (**Figure 4K and 4L**). Above findings demonstrate that USP7 directly binds to SMAD3, and this interaction is mediated by the UBL domain of USP7. Similarly, we constructed 3 mutant plasmids of SMAD3 (MH1, Linker and MH2 domain) and found that SMAD3 interacted with USP7 through MH2 domain (**Figure S13**).

### USP7 regulates the stability of SMAD3 protein through deubiquitination process

**Figure 5:**
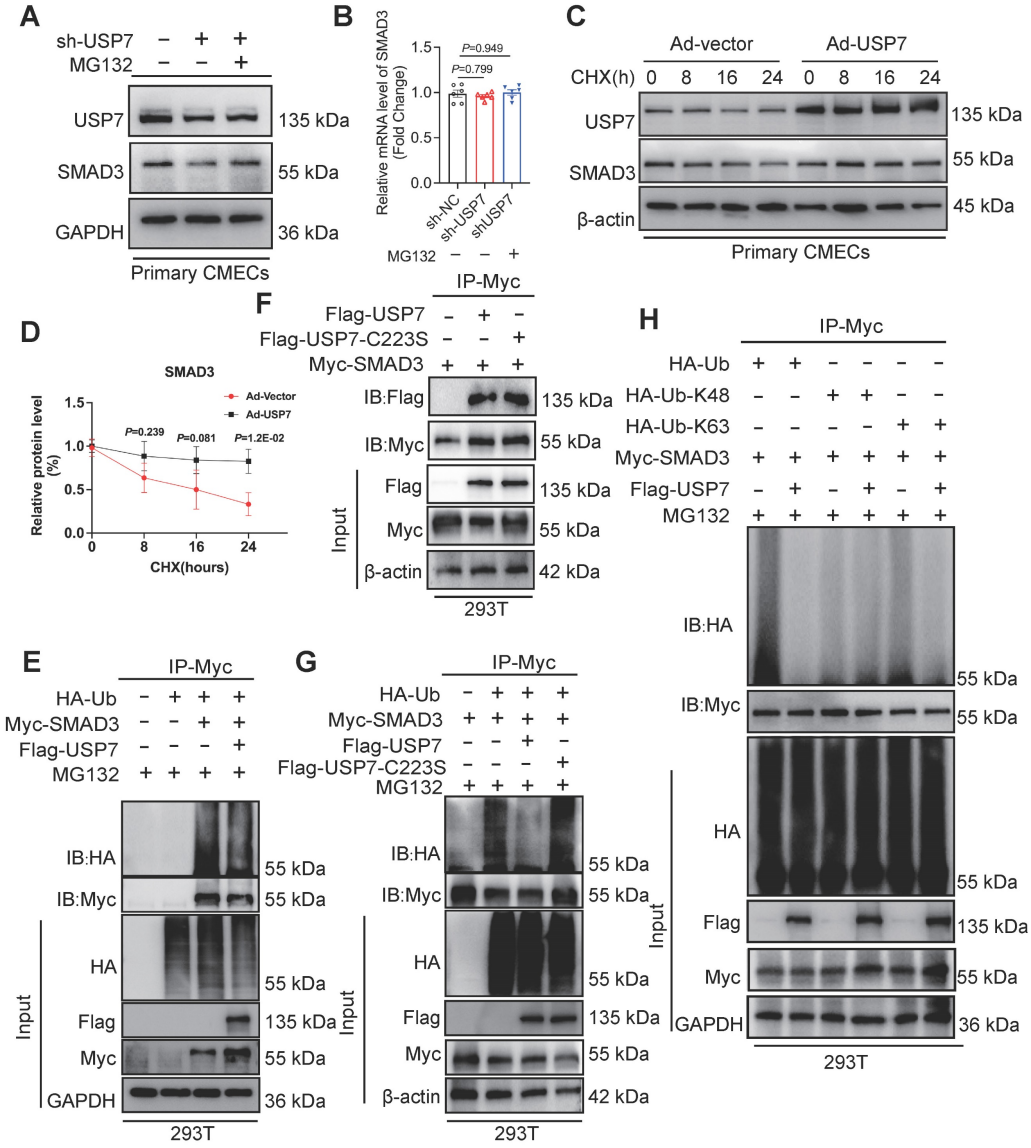
** USP7 regulates the stability of SMAD3 protein through deubiquitination. A** and** B**, Representative western blotting (**A**) and real-time qPCR (**B**) for USP7 and SMAD3 in CMECs transfected with short hairpin RNA lentiviral particles targeting USP7 (shUSP7) or control adenovirus (sh-Vector), and treated for MG132 or PBS for 4 hours. n = 6. **C and D**, Representative western blotting for USP7 and SMAD3 in CMECs co-transfected with control adenovirus or recombinant USP7 adenovirus and then subjected to CHX pulse-chase assay (**C**) and densitometric quantification of SMAD3 (**D**). n = 4. **E**, Western blot analysis of indicated proteins in 293T cells cotransfected with Myc-SMAD3 and HA-Ub in the presence of Flag-vector or Flag-USP7 plus the proteasome inhibitor MG132 (10 μM) for 4 hours before IP of whole cell lysates with MYC magnetic beads (n = 3). **F**, Coimmunoprecipitation of USP7 and SMAD3 in 293T cells co-transfected with Flag-USP7 or Flag-USP7-C223S and Myc-SMAD3 plasmids. Exogenous SMAD3 was immunoprecipitated by anti-Myc antibody, n = 3. **G**, Western blot analysis of indicated proteins in 293T cells cotransfected with Myc-SMAD3 and HA-Ub in the presence of Flag-vector, Flag-USP7 or Flag-USP7-C223S plus the proteasome inhibitor MG132 (10 μM) for 4 hours before IP of whole cell lysates with MYC magnetic beads (n = 3). **H,** Western blot analysis of indicated proteins in 293T cells cotransfected with Myc-SMAD3 and HA-Ub, HA-K48 and HA-K63 in the presence of Flag-vector or Flag-USP7 plus the proteasome inhibitor MG132 (10 μM) for 4 hours before IP of whole cell lysates with MYC magnetic beads (n = 3). **B**: 1-way ANOVA followed by Tukey post-hoc tests; **D**, 2-way ANOVA plus Šídák's multiple comparisons test; Data are shown as mean±SEM and adjusted P values were provided in case of multiple groups. The protein level was standardized by GAPDH or β-actin and the mRNA level from each group was normalized to 1 value from the control group, which was set to 1.

The interaction and regulatory relationship between USP7 and SMAD3 raise the question of how USP7 regulates SMAD3. Interestingly, we observed a decrease in SMAD3 protein levels upon reducing USP7 expression, and this decrease was not attributed to altered transcription of SMAD3 (**Figure 5A and 5B**). This suggests that USP7 may regulate SMAD3 at the post-translational level, possibly through protein stabilization or degradation pathways. We hypothesized that USP7 might prevent the intracellular degradation of SMAD3. In fact, cycloheximide (CHX) pulse-chase assay revealed that USP7 obviously prevented SMAD3 from proteasomal degradation (**Figure 5C and 5D**). This indicated that USP7 might play a role in mitigating degradation of SMAD3 protein. It is known that USP7 might function as a deubiquitylating enzyme, exert its regulatory role by removing ubiquitin molecules from target proteins. The exact regulatory mechanism of USP7 in SMAD3 ubiquitination was explored. Myc-SMAD3 and ubiquitin plasmids were co-transfected into 293T cells. Subsequently, the transfected cells were divided into two groups: one group was transfected with Flag-USP7 plasmid, while the other group was transfected with Flag-vector plasmid as a control.

Then, the cells were treated with MG132 to inhibit proteasomal degradation of the SMAD3 protein. Notably, a significant decrease was observed in the presence of ubiquitin molecules on SMAD3 in cells transfected with the Flag-USP7 plasmid compared to cells transfected with the Flag-vector plasmid (**Figure 5E**). It has been reported that cysteine at position 223 was crucial for USP7 to exert its deubiquitination function^27^. To further identify the deubiquitination sites of USP7, a catalytic mutant USP7 (C223S) was constructed. Results showed that the catalytic mutant USP7 (C223S) failed to reduce the ubiquitination level of SMAD3, and could not affect the binding of them (**Figure 5F and 5G**). To investigate the specific ubiquitin chains recognized by USP7, we further generated ubiquitin plasmids retain only K48 or K63 active sites. Results showed that USP7 primarily exerts its deubiquitination function by recognizing and cleaving ubiquitin chains at the K63 sites (**Figure 5H**). These results thus indicate that USP7 could remove K63-linked ubiquitin molecules from SMAD3 and prevent SMAD3 from proteasomal degradation, and the cysteine at position 223 of USP7 is implicated in the removal of ubiquitin molecules from SMAD3, thereby preventing its degradation.

### USP7 regulates EndMT by stabilizing SMAD3 and accumulating phosphorylated SMAD3

**Figure 6:**
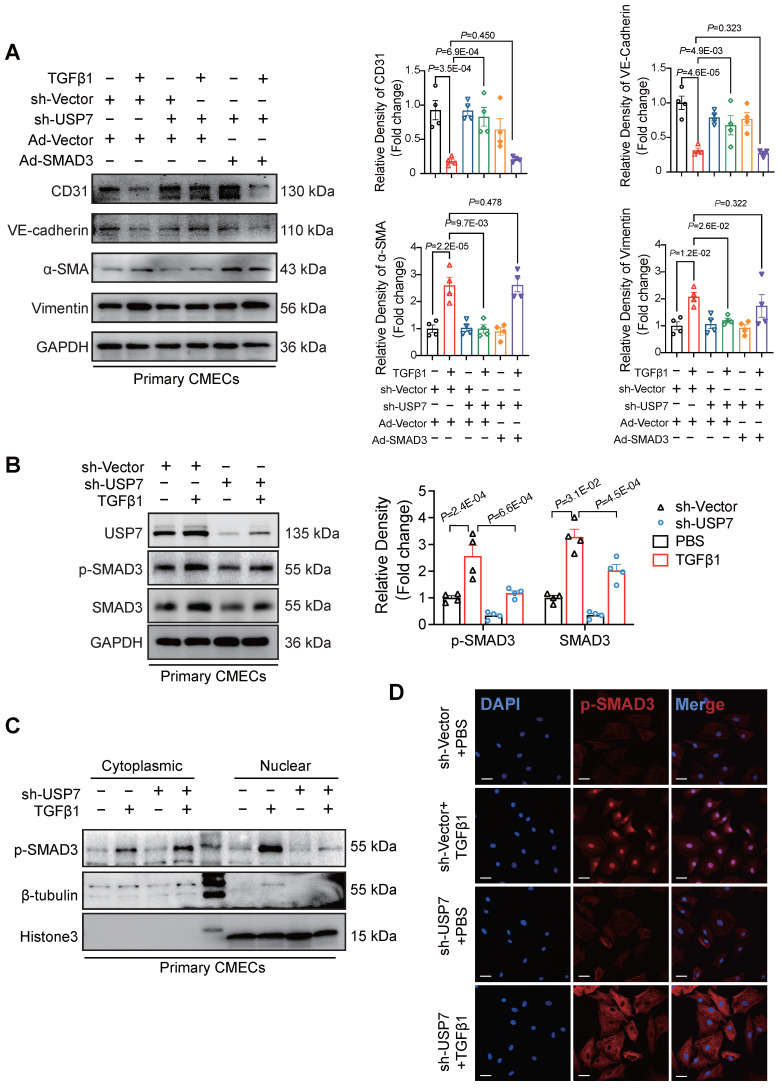
** USP7 Regulates EndMT by stabilizing SMAD3 and accumulating phosphorylated SMAD3. A**, Representative western blotting analysis and densitometric quantification of endothelial cell marker (CD31, VE-cadherin), and mesenchymal marker (α-SMA, Vimentin) in primary cardiac microvascular endothelial cells (CMECs) cotransfected with short hairpin RNA lentiviral particles targeting USP7 (shUSP7)/control adenovirus (sh-Vector) and control adenovirus (Ad-Vector)/recombinant SMAD3 adenovirus (Ad-SMAD3) under TGFβ1 stimulation (10 ng/ml, 72h) or PBS control. n = 4. **B**, Representative western blotting analysis (**C**) and densitometric quantification (**D**) of p-SMAD3 and SMAD3 protein level in primary cardiac microvascular endothelial cells (CMECs) transfected with short hairpin RNA lentiviral particles targeting USP7 (shUSP7)/control adenovirus (sh-Vector) under TGFβ1 stimulation (10 ng/ml, 72h) or PBS control. n = 4. **C**, Representative western blotting analysis of p-SMAD3 and SMAD3 protein level in cytoplasm and nucleus in primary cardiac microvascular endothelial cells (CMECs). β-Tubulin was used as the loading control for cytosolic fractions. Histone 3 was used as the loading control for nuclear fractions. n = 4. **D,** Immunofluorescence staining of SMAD3 (red) and DAPI (blue) in primary cardiac microvascular endothelial cells (CMECs) transfected with short hairpin RNA lentiviral particles targeting USP7 (shUSP7)/control adenovirus (sh-Vector) under TGFβ1 stimulation (10 ng/ml, 72h) or PBS control. Scale bars, 20 μm. **A, B**: 1-way ANOVA followed by Tukey post-hoc tests; Data are shown as mean±SEM and adjusted P values were provided in case of multiple groups. The protein level was standardized by GAPDH from each group was normalized to 1 value from the control group, which was set to 1.

To further define the mechanistic role of SMAD3 in the USP7-mediated EndMT process, effects of overexpressing SMAD3 while simultaneously knocking down USP7 were observed in CMECs. The results indicated that the improvement in the EndMT phenotype resulting from the knockdown of USP7 was partially attenuated by overexpressing SMAD3 (**Figure 6A, Figure S14**). The process of SMAD3 activation contains phosphorylation and nuclear translocation. We demonstrated that down-regulation of USP7 reduced the protein level of SMAD3 in the previous part. However, whether this down-regulation could indirectly lead to the decrease of phosphorylated SMAD remains unclear. Therefore, we then explored whether USP7 could modulate the levels of phosphorylated SMAD3 (p-SMAD3). Results showed that knocking down USP7 resulted in a reduction of the elevated levels of pSMAD3 induced by TGFβ1 (**Figure 6B**). Interestingly, further nuclear-cytoplasmic separation assays and immunofluorescence staining experiments provided additional evidence in that knocking down USP7 impeded the translocation of pSMAD3 into the nucleus (**Figure 6C and 6D**). These *in vitro* study results thus demonstrate that USP7 could regulate the phosphorylation and nuclear translocation of SMAD3, ultimately modulate the EndMT process in CMECs stimulated by TGFβ1.

### USP7 knocking out ameliorates cardiac fibrosis and EndMT of HFpEF via SMAD3-dependent pathway *in vivo*

**Figure 7:**
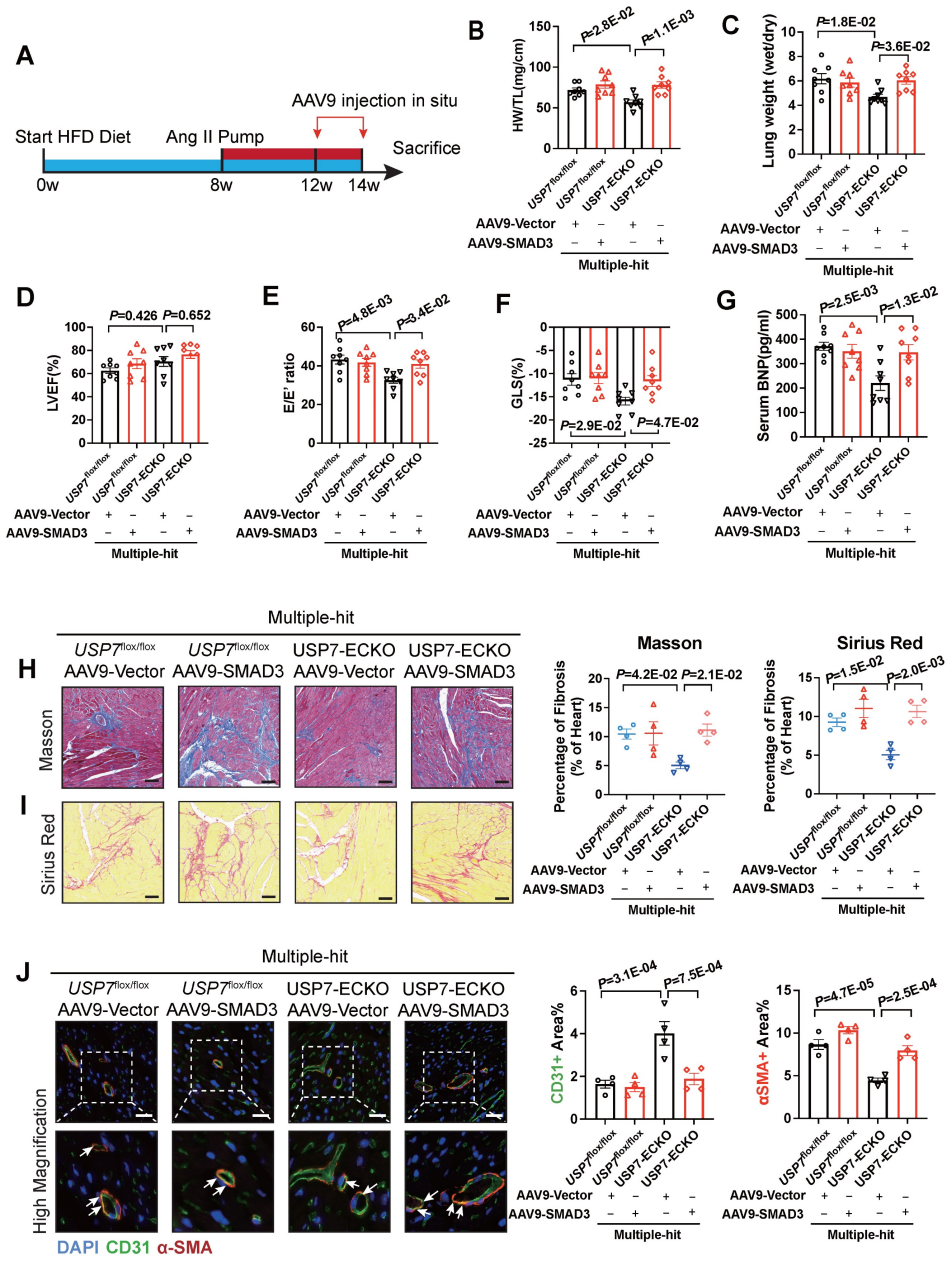
** USP7 ameliorates cardiac fibrosis and EndMT of HFpEF by stabilizing SMAD3 *in vivo*. A**, Schematic of the experimental setup. After “Multiple-hit” strategy for 12 weeks, recombinant AAV9-ENT vectors carrying SMAD3 or ctrl were injected to heart of mice *in situ* for 2 weeks. **B**, Heart weight (HW) normalized to tibia length (TL). **C**, Ratio between wet and dry lung weight. **D**, Percentage of left ventricular ejection fraction (LVEF). **E**, Ratio between mitral E wave and E' wave (E/E'). **F**, Percentage of global longitudinal strain (GLS). **G**, The serum levels of NT-proBNP (N-terminal pro-B-type natriuretic peptide). **H** and **I**, Representative and quantification in Masson (**B**) and Sirius Red (**C**) staining in sections of hearts. (Scale bar, 100 μm for Masson; 100 μm for Sirius Red staining). **J**, Representative immunofluorescent staining images and quantification of microvascular endothelial cell CD31 (green) and fibrosis marker α-SMA (red) in the heart tissues (n = 4). Scale bar, 20 μm. **B-J**: 1-way ANOVA followed by Tukey post-hoc tests; Data are shown as mean±SEM and adjusted P values were provided in case of multiple groups.

To validate the role of SMAD3 in USP7 knocking out-mediated beneficial effects in this HFpEF model, we constructed AAV9-ENT vectors (based on adeno-associated virus 9 (AAV9) serotype modification and enhanced the infection efficiency of vascular endothelial cells) carrying SMAD3 under the ICAM2 promoter. And then delivered these AAV9-ENT vectors via cardiac injection *in situ* (**Figure 7A**). We confirmed that SMAD3 was highly expressed in cardiac ECs of HFpEF mice (**Figure S15**). Injection of AAV9-ENT did not have a significant effect on weight gain and blood pressure following the “Multiple-hit” strategy in mice (**Figure S16**). The heart weight-to-tibial length ratio (HW/TL), lung weight/dry ratio, and exercise tolerance measurements indicated that myocardial overexpression of SMAD3 in EC-specific USP7 knockout mice reversed the protected effects of knocking out USP7 in the HFpEF phenotypes induced by the “Multiple-hit” strategy (**Figure 7B and 7C**). Additionally, it was observed that myocardial overexpression of SMAD3 in EC-specific USP7 knockout mice reversed the protective effects of EC-specific USP7 knockout in terms of cardiac function, fibrosis, and EndMT post the “Multiple-hit” strategy (**Figure 7D through 7F, Figure S16**). Serum BNP levels changed in line with above changes (**Figure 7G**).

## Discussion

**Figure 8:**
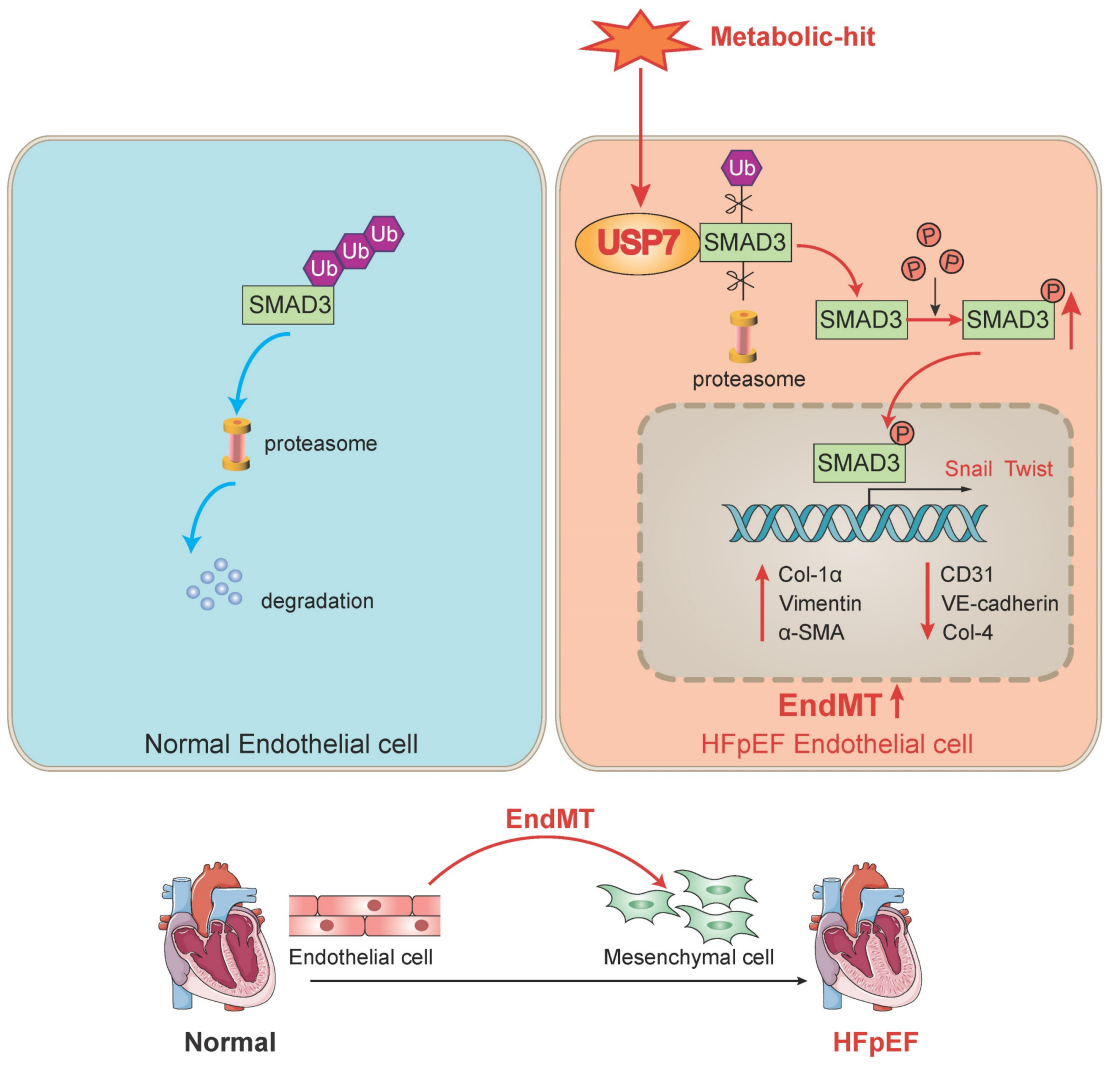
** A schematic diagram of this study.** In normal endothelial cells, SMAD3 undergoes ubiquitination and normal degradation. However, under various metabolic-hit conditions, the abnormal increasing USP7 leads to a reduction in SMAD3 ubiquitination and degradation. This, in turn, promotes the activation of SMAD3, facilitating its entry into the nucleus and promoting the EndMT process. Consequently, this cascade of events contributes to cardiac fibrosis and HFpEF. Ub, ubiquitin; EndMT. endothelial-to-mesenchymal transition; HFpEF, heart failure with preserved ejection fraction.

Our study revealed that USP7 plays a pivotal role in the progression of cardiac fibrosis in HFpEF mice by promoting the process of EndMT. The novel findings of our study are as follows: 1. upregulation of USP7 and SMAD3 was identified in the cardiac microvascular endothelial cells of the “Multiple-hit” HFpEF mouse model; 2. EC-specific knockout of USP7 significantly ameliorated cardiac diastolic dysfunction, reduced cardiac fibrosis, and mitigated EndMT in HFpEF mice; 3. Mechanistically, USP7 could remove K63-linked ubiquitin molecules from SMAD3 and prevent SMAD3 from proteasomal degradation, and the cysteine at position 223 of USP7 was implicated in the removal of ubiquitin molecules from SMAD3, thereby preventing its degradation, USP7 thus enhanced the stability of SMAD3 and regulated the phosphorylation and nuclear translocation of SMAD3, thereby facilitating the transcription of genes related to EndMT. Collectively, our study demonstrated knocking out USP7 could ameliorate diastolic dysfunction and reduce cardiac fibrosis through promoting the degradation of SMAD3 in HFpEF, primarily by mitigating the process of EndMT (**Figure 8**). Inhibiting USP7 and SMAD3 might be feasible ways to alleviate HFpEF pathology.

The development of effective, evidence-based treatments for HFpEF is challenged by the phenotypic heterogeneity and the complexity of underlying pathogenesis of HFpEF^28^, ^29^. One important obstacle is the absence of the animal model that accurately replicates the complexities of human HFpEF^30^, ^31^. Patients with HFpEF usually present with multiple comorbidities, including obesity, hypertension, diabetes, and other metabolic disorders^3^, ^32^. Several research groups developed animal models for HFpEF, each of them with distinctive strengths and limitations. The efficacy of animal models could be partially evaluated in the two recent HFpEF scoring systems^33^, ^34^. In our study, the animal model got a score of 4 points in the H2PEF scoring system and 5 points in the HFA-PEF scoring system, which was consistent with the results of previous study^24^. This suggests that the HFpEF model used in this study resembles the human clinical situation to some extent.

EndMT is the process in which endothelial cells (ECs) transform into mesenchymal cells. This phenomenon is implicated in a variety of cardiovascular diseases, including valve disease, myocardial infarction (MI), fibrosis, endocardial fibroelastic fibrosis, atherosclerosis, and pulmonary arterial hypertension (PAH)^14^. A recent study demonstrated that *in vitro* cultivation of human aortic endothelial cells using serum obtained from patients with HFpEF could stimulate endothelial EndMT and there is thus a close clinical association between EndMT and HFpEF^35^. In line with this finding, our results hinted that alleviated HFpEF phenotypes was related to reduced EndMT in our HFpEF model post USP7 knocking out.

DUBs can modify the signal transmission and protect substrate proteins from degradation by regulating the form of ubiquitin molecule linkage. Understanding the regulatory mechanisms of DUBs in HFpEF is anticipated to yield novel therapeutic strategies. Previous studies have also demonstrated that DUBs are implicated in a range of cardiovascular diseases, including cardiac hypertrophy^36^, ^37^, cardiomyopathy^38^, and vascular remodeling^39^. However, as far as our knowledge extends, there were no prior studies investigating the functioning of DUBs in HFpEF. Our study revealed significant upregulation of USP7 in the cardiac microvascular endothelium of HFpEF mice, and EC-specific knockout of USP7 improved HFpEF phenotypes, including cardiac diastolic function, myocardial fibrosis, and exercise tolerance in HFpEF mice, indicating a crucial role of USP7 in the pathogenesis of HFpEF.

The role of DUBs is closely related to the function of the substrate proteins. Through LC-MS/MS, we identified SMAD3 as a substrate of USP7. This finding was further confirmed through co-immunoprecipitation (Co-IP) experiments. The TGFβ-SMAD3 signaling pathway serves as a primary inducer of EndMT, SMAD3 is activated through phosphorylation, subsequently translocating into the nucleus to regulate the transcription of proteins associated with EndMT^40^. Previous studies demonstrated that the specific knockdown or inhibition of SMAD3 could effectively mitigate EndMT^41^, ^42^. Ubiquitination is one of the key mechanisms involved in the degradation of SMAD3^43^. Simultaneously, DUBs possess the capability to inhibit the ubiquitination and subsequent degradation of SMAD3, enabling precise regulation of SMAD3 levels. OTUB1, UCHL5, OTUD1 and USP15 have been reported to participate in the regulation of SMAD3 deubiquitination, and the abnormal expression of DUBs disrupts the dynamic balance of SMAD3, consequently promoting the development of various pathological processes^39^, ^44^-^46^. It had been reported that USP7 could influence the progression of p53-negative lung cancer by regulating SMAD3^47^. In our study, we demonstrated that the abnormal activation of USP7 might not lead to beneficial effects in HFpEF. Due to the abnormal activation of USP7, SMAD3 was upregulated, which in turn increased the EndMT process of ECs, promoted myocardial fibrosis, and accelerated the progression of HFpEF. Inhibiting the abnormal activation of USP7 in ECs might be an effective target to improve the prognosis of HFpEF. This suggested that USP7 might play different roles in different diseases and cell types. Our study complemented the existing research by addressing the role of USP7 in the field of HFpEF. In addition, our study also showed that during EndMT in CMECs, USP7 primarily influenced the post-translational modification process. USP7 removed K63-linked ubiquitin molecules from SMAD3, preventing its proteasomal degradation. Our research enriched the understanding of regulatory mechanism between USP7 and SMAD3.

Briefly, we identified USP7 as a crucial DUB that regulates SMAD3 and play pivotal role in the EndMT process of HFpEF cardiac fibrosis. Our results showed that USP7 sustained SMAD3 stability via promoting deubiquitination of SMAD3, reversing K63-linked ubiquitin chains in the cysteine at position 223 of USP7. Furthermore, USP7 also enhanced the activation of SMAD3 by promoting SMAD3 phosphorylation. Identifying the specific active site of USP7 that regulates SMAD3 ubiquitination can streamline drug development targeting this specific site, without impacting the functions of substrate proteins at other active sites of USP7, and thereby minimizing potential drug side effects.

Some limitations of this study should be acknowledged. First, the improvement in the HFpEF phenotypes achieved through EC-specific knockout of USP7 may not solely be attributed to the regulation of SMAD3 to alleviate EndMT. Other potential mechanisms may also be involved, as evidenced by the reduced plasma TGFβ1 levels observed in mice with EC-specific knockout of USP7 (**Figure S7J**). Second, *in vitro* experiments may not entirely replicate the conditions under which EndMT occurs in the complex "Multiple-hit" state of the HFpEF phenotypes. Additionally, there may be other pathways contributing to the process of EndMT in our HFpEF model, such as metabolic transitions. Further investigations are also required to explore if there is an add-on effects on HFpEF by jointly inhibition of USP7 and SMAD3. Third, because of the challenges in acquiring myocardial tissue samples from HFpEF patients, our dataset lacked validation from patient myocardial tissue. Further validation of the HFpEF phenotypes in humans would enhance the clinical translational significance of this study.

In conclusion, our study unveiled the pivotal role of USP7 in endothelial cells as a key regulator of EndMT in the context of HFpEF. EC-specific knockout of USP7 could ameliorate cardiac diastolic function, reduce cardiac fibrosis, and mitigate EndMT in HFpEF mice in a SMAD3 dependent pathway. Our studies hinted that inhibiting USP7 and SMAD3 alone or in combination might be potential therapeutic option for the management of HFpEF.

## Supplementary Material

Supplementary materials and methods, figures and tables.

## Abbreviations

Ang II: angiotensin II
ARNI: angiotensin receptor-neprilysin inhibitor
BNP: B-type natriuretic peptide
CHX: cycloheximide
CMECs: cardiac microvascular endothelial cells
Co-IP: co-immunoprecipitation
DUBs: deubiquitinating enzymes
ECM: extracellular matrix
ECs: endothelial cells
EMT: epithelial-mesenchymal transition
EndMT: endothelial-mesenchymal transition
GLS: global longitudinal strain
HF: heart failure
HFpEF: heart failure with preserved ejection fraction
HFrEF: heart failure with reduced ejection fraction
HW/TL: heart weight-to-tibia length ratio
IVRT: isovolumic relaxation time
LVEF: left ventricular ejection fraction
LVPWd: left ventricular posterior wall thickness in diastole
MI: myocardial infarction
NO: nitric oxide
PAH: pulmonary arterial hypertension
p-SMAD3: phosphorylated SMAD3
SGLT2i: sodium-glucose cotransporter 2 inhibitor
USP7: ubiquitin-specific protease 7
