# Supplementary material for: Knocking out USP7 attenuates cardiac fibrosis and endothelial-to-mesenchymal transition by destabilizing SMAD3 in mice with heart failure with preserved ejection fraction
Source: Theranostics. 2024 Sep 9;14(15):5793–808. doi: 10.7150/thno.97767 (PMC11426239; doi:10.7150/thno.97767)
Supplement: Supplementary file 1 — Supplementary materials and methods, figures and tables. [file thnov14p5793s1.pdf]

SUPPLEMENTAL MATERIAL

Knocking down USP7 Attenuates Cardiac Fibrosis and Endothelial-to-Mesenchymal Transition by Destabilizing SMAD3 in Mice With Heart Failure with Preserved Ejection Fraction

Shuai Yuan, etc.

Running title: Knocking down USP7 Attenuates HFpEF

#Correspondence to: Jingmin Zhou, MD, PhD, and Junbo Ge, MD, PhD, Department of Cardiology, Shanghai Institute of Cardiovascular Diseases, Zhongshan Hospital, Fudan University, 180 Fenglin Road, Xuhui District, Shanghai, 200032, China.

E-mail: zhou.jingmin@zs-hospital.sh.cn; jbge@zs-hospital.sh.cn

This file includes:

1. Supplemental Materials and Methods
2. Supplemental Figure 1-16;
3. Supplemental Table 1-5.

Supplemental Materials and Methods

Regents

Ang II (A9525) was purchased from Sigma-Aldrich (St. Louis, USA). TGFβ1 (RP00161) were purchased from ABclonal (Wuhan, China). The overexpression vector and adenovirus of Ad-USP7, Ad-Smad3, short hairpin RNA lentiviral particles targeting USP7 (shUSP7) and non-targeted short hairpin RNA (AdshRNA) were obtained from HANBIO (Shanghai, China). Plasmids (Flag-USP7, Myc-Smad3, HA-Ub, HA-K48, HA-K63) and small interfering RNA against Smad3 and scrambled sequences were obtained from Genomeditec (Shanghai, China). Primary antibodies used in this study include：Antibodies against USP7 (#4833) , Mouse Anti-Rabbit IgG (Light-Chain Specific) (#93702) , Rabbit Anti-Mouse IgG (Light Chain Specific) (#58802) were purchased from Cell Signaling Technology (MA, USA); VE-cadherin(A22659), Snail(A11794), Smad3(A19115), GAPDH(AC035) , β-actin (AC028) were purchased from ABclonal (Wuhan, China). pSmad3 (ab52903), CD31(ab9498), Alexa Fluor® 488 (ab150113) and Alexa Fluor® 594 (ab150080) were purchased from Abcam (Cambridge, UK); α-SMA (14395-1-AP), Vimentin (10366-1-AP), Flag tag (20543-1-AP), Myc tag (16286-1-AP), HA tag (51064-2-AP) were purchased from Proteintech (Hubei, China). Goat anti-Rabbit IgG (SPA134), the Masson's Trichrome Stain Kit (G1340) and Picrosirius Red (S8060) were purchased from Solarbio Life Sciences (Beijing, China).

Animal model

Generation of endothelial specific USP7 knockout mice

The mouse studies followed standard laboratory protocols for randomization. All animal care and

experimental procedures were approved by the Animal Care and Use Committee of Zhongshan Hospital, Fudan University. All studies conducted were in accordance with ethical regulations and guidelines. *USP7*$^{flox/flox}$ mice and Cdh5-Cre^{ERT} mice on C57BL/6J background, aged 8 - 10 months, were purchased from Cyagen (Suzhou, China). Endothelial-specific conditional USP7 deficiency mouse (*USP7*$^{flox/flox}$/Cdh5-Cre^{ERT}) were generated by crossing *USP7*$^{flox/flox}$ mice with Cdh5-Cre^{ERT} mice and intraperitoneally injected with tamoxifen (30 mg/kg) daily for 5 days. All the mice were placed in a temperature-controlled cage with 12-hour light and dark cycles at 22 ± 2°C and were given free access to food and water.

Experimental HFpEF mouse model

In HFpEF group, female, 18- to 22-month-old C57B6/J mice were fed a high-fat diet (HFD, 60% kcal fat; Research Diets D17041409) for 12 weeks. And after 8 weeks of diet, the mice underwent surgery under anesthesia, during which a surgical subcutaneous pocket was created in the back of each mouse. An ALZET® osmotic mini pump was implanted, and the mice were subjected to continuous ANGII (1.25 mg/kg/day) (Sigma) administration for a duration of 4 weeks. Mice in control group were female, 18- to 22-month-old and provided with a standard normal diet (20% kcal fat, Research diets D17041407) and had surgical pockets created in their backs at week 8, but no mini pump was implanted. The sample size of mice experiments was determined based on the mean and standard deviation obtained from previous literature reports and pre-experimental findings. The minimum sample size needed for each group was 5 with alpha level = 0.05 and power = 0.8. Thus, based on our calculations, a sample size of 6 to 10 mice per group will be sufficient for our experiment. Echocardiography and Doppler imaging were conducted at the end of the 12th week. The mice were sacrificed to collect serum and heart tissue samples at the end of the experiment. The

researchers adhered to standard laboratory procedures for randomization and conducted data analysis in a blinded manner. No samples or animals were intentionally excluded from the analyses. In animal studies, the investigators were blind to treatment/genotype group during the experiment and quantification. However, blinding was not implemented in the in vitro cell experiments.

Echocardiography

The cardiac dimensional and functional parameters were assessed using transthoracic ultrasound, as described previously[1] (Visual Sonics Vevo 2100 Imaging System, Toronto, Canada). Mice were administered a gas mixture comprising 2% - 3% isoflurane and oxygen through a mask to induce anesthesia. After completing all procedures, all mice recovered with no difficulties.

Exercise exhaustion test

After three days of acclimating to the treadmill (Yuyan Instruments Co, Shanghai, China), an exhaustion test was conducted following the procedures described previously[2]. The treadmill was initiated at a speed of 10 m/min with an inclination of 0°. The inclination was then increased by 5° every 5 minutes until it reached a maximum of 30°. The speed was augmented by 1 m/min every 3 minutes until the last mouse reached exhaustion. Exhaustion was defined as the mice's inability to resume running within 10 seconds after being directly exposed to the electrical stimulation network. The total length and distance of each mouse's run were recorded during the experiment.

Blood pressure measurement

Blood pressure was monitored using the standard noninvasive tail-cuff method (BP-2000; Visitech

Systems). The mice were placed in a box with their tails threaded through a compression cuff equipped with optical sensors and positioned on a heated platform. Blood pressure measurements were taken 20 minutes after the mice had acclimated. Each mouse underwent 20 practical measurements, with at least 60% of successful measurements included for analysis. The average of the successful blood pressure measurements was recorded as the blood pressure of mice.

ELISA

Mouse plasma samples were tested using an ELISA kit following the manufacturer's instructions. The serum B-type natriuretic peptide (BNP) of mice was detected using the Mouse BNP ELISA Kit (Cat No. SEKM-0151, Solarbio, Beijing, China). The serum TGFβ1 of mice was measured using the Mouse TGF-β1 ELISA Kit (Cat No. SEKM-0035, Solarbio, Beijing, China).

Isolation of primary neonatal rat cardiac microvascular endothelial cells (CMECs)

The method of primary rat CMECs isolation is in accordance with the procedure outlined in our previous work[3]. 2-week-old male SD rats weighing 30 ± 5g were euthanized, and their hearts were rapidly excised and washed in 4°C pre-cooled PBS. Subsequently, the atrial muscle and large blood vessels were removed, and the left ventricle was incised to eliminate the endocardium and epicardium. The remaining ventricular tissue was sectioned into 1mm^3 pieces and evenly distributed in a 10 cm culture dish pre-coated with 1ml of FBS (fetal bovine serum). The tissue pieces were cultured in a humidified tissue incubator (37°C, 5% CO_2) for 4 hours. Once the tissue pieces were fully attached to the cell culture dish, 6ml of DMEM medium containing 10% FBS was added, and the cultures were continued in a humidified tissue incubator (37°C, 5% CO2) for 48 hours until a

significant number of polygonal or star-like cells crawled out from the myocardia. When the distribution of endothelial cells (ECs) reached 80%, the tissue mass was washed away, and the cells were detached using 0.25% trypsin for passaging. The second generation of cells was utilized for the experiment.

Isolation of primary cardiomyocytes from HFpEF mice

Adult primary cardiomyocytes were isolated from the ventricles of mice and utilized directly for further experiments, following the procedures described in previous reports[4]. The mice were euthanized, and the descending aorta was severed. The right ventricle was injected with EDTA buffer to flush the blood. Subsequently, the heart was transferred to a 6 cm cell dish, and the left ventricle (LV) was sequentially injected with EDTA buffer, perfusion buffer, and collagenase buffer for digestion. The heart is separated and dissected into small 1 mm^3 pieces. The enzyme activity is subsequently halted by treating the tissue with a stop buffer. The cell suspension was filtered through a 100 μm filter, and four rounds of gravity sedimentation were conducted using three intermediate calcium reintroduction buffers, gradually restoring the calcium concentration to physiological levels. The cardiomyocytes were resuspended in preheated media and distributed according to the desired application density. They were then cultured in a humidified tissue incubator at 37°C with 5% CO_2, in media pre-coated with Laminin (5 μg/ml). After 1 hour, the medium was replaced with fresh medium, and the cardiomyocytes were then used in the experiment.

Isolation of primary cardiac ECs from HFpEF mice

The mice were euthanized, and the interior of the heart was rinsed with cold phosphate-buffered

saline (PBS). The heart was then cut into 2 mm pieces and digested with an enzyme kit (Cat No. 130-110-201, Miltenyi Biotec) following the instructions to obtain a single-cell suspension. The obtained single-cell suspension was filtered using a 70 μm filter to remove cell clumps. Subsequently, CD31 MicroBeads (Cat No. 130-077-418, Miltenyi Biotec) at a concentration of 1×10^7 total number of MicroBeads per 10μL were added to the cell suspension and incubated at 4°C for 15 minutes. The cells were washed, and then the isolated primary cardiac endothelial cells (ECs) were processed using the LD column (Cat No. 130-042-901, Miltenyi Biotec) following the manufacturer's instructions. The isolated ECs were immediately utilized for further experiments.

Cell culture and transfection

HEK 293T cells (Cat No. SCSP-502) were procured from the Shanghai Institute of Biochemistry and Cell Biology (Shanghai, China). Isolated CMECs were culture in high-glucose DMEM, which contains 10% fetal bovine serum and 1% penicillin/streptomycin. When the cell density in the 6-well plate reached 60%, 1 μg of plasmid was transfected using Opti-MEMTM Medium (cat. no. 31985070, Thermo Fisher Scientific, Germany), along with 2 μL of Lipofectamine^{TM}3000 and 2 μL of P3000 (Cat. No. L3000-015, Thermo Fisher Scientific, Germany). Additionally, 50 nM of siRNA was transfected using Opti-METM medium containing 2 μL of Lipofectamine^{TM}3000. The cells were transfected 24 hours later to proceed with the follow-up experiment. Isolated cardiomyocytes were culture in high-glucose DMEM, which contains 10% fetal bovine serum. Isolated cardiac ECs were cultured in endothelial cell medium (Cat No. 1001, ScienCell Research) comprising basal endothelial cell medium, endothelial cell growth supplement, penicillin/streptomycin solution, and fetal bovine serum (5%). To induce overexpression of USP7/Smad3, CMECs were infected with

control adenovirus or recombinant USP7/SMAD3 adenovirus (HANBIO, Shanghai, China). Short hairpin RNA lentiviral particles targeting USP7 (shUSP7, HANBIO, Shanghai, China) were employed to down-regulate USP7 expression in CMECs. As a control, non-targeted short hairpin RNA (AdshRNA) was inserted into the same adenovirus vector.

Histological assessment

The heart tissue was fixed with 4% formaldehyde, dehydrated, and made transparent. Subsequently, the tissue was embedded in paraffin. Finally, the heart tissue was sectioned into 5 μm thick samples for further experimental analysis. The tissue sections were stained with Masson stain (Cat No. G1340, Solarbio, Beijing, China) and Picrosirius Red (Cat No. S8060, Solarbio, Beijing, China) according to the manufacturer's instructions. For immunofluorescence staining, tissue sections were infiltrated with 0.1% Triton X-100 for 10 minutes and then blocked with 5% BSA for 1 hour. The samples were then incubated with the primary antibody at 4°C overnight. On the second day, the sections were incubated with the secondary antibody coupled with a fluorophore. Finally, the slides were counterstained with DAPI for 5 minutes. Immunofluorescence staining of cell samples followed a similar procedure, except that the cells were fixed with 4% paraformaldehyde for 15 minutes and permeabilized with 0.5% Triton X-100 for 10 minutes. Images were acquired using the Olympus laser confocal microscope (Olympus FV3000, Japan).

Adeno-associated virus serotype 9 (AAV9) injection

To further investigate the role of USP7 and SMAD3 in HFpEF cardiac fibrosis and EndMT, we constructed AAV9-ENT vectors (based on adeno-associated virus 9 (AAV9) serotype modification

and enhanced the infection efficiency of vascular endothelial cells) carrying SMAD3 under the ICAM2 promoter. And then delivered these AAV9-ENT vectors via cardiac injection in situ and empty vector (AAV9-NC) (HANBIO, Shanghai, China) and performed injections into the hearts of 4 groups of mice in situ (2×10^{11} particles/mouse). Euthanasia was conducted 2 weeks after the injection, and the expression efficiency was confirmed through western blot and immunofluorescence staining.

RNA-sequencing

Total RNA from the heart tissues of HFpEF mice and normal aging wild-type mice was isolated using TRIZOL reagent (Cat No.15596026; Thermo Fisher). RNA integrity was assessed by denaturing agarose gel electrophoresis. Subsequently, the Poly(A) RNA is fragmented into small pieces, and cDNA synthesis is performed using SuperScript II reverse transcriptase (Cat No. 18064014, Thermo Fisher). After pretreatment, the ligation products were subjected to PCR amplification. The average size of the cDNA library was 300 ± 50 bp. Finally, 2×150bp end-to-end sequencing (PE150) was carried out using the Illumina Novaseq 6000 (LC-BioTechnology Co, Ltd in Hangzhou, China) following the instructions.

Quantitative Real-time Polymerase Chain Reaction (RT-qPCR)

Total RNA was extracted from animal heart tissues and CMECs using the UNIQ-10 Column Trizol Total RNA Isolation Kit (Cat No. B511321, Sangon Biotech, Shanghai, China), following the provided instructions. mRNA was reverse-transcribed into cDNA using the PrimeScript RT Reagent Kit (Cat No. RR036A, TAKARA, Janpan). Real-time quantitative PCR(RT-qPCR) was performed

using SYBR Green Master Mix (Cat No. 11198ES03, Yeason, Shanghai, China). All RT-qPCR experiments were repeated at least 3 times. The primer sequences used for qRT-PCR and cDNA cloning was shown in supplemental data Table S4 and S5.

Western blotting and co-immunoprecipitation

Proteins in mice myocardial tissues and experimental cells were extracted with RIPA buffer (P0013B, Beyotime Biotechnology, Shanghai, China). Equal amounts of protein were added, separated by sodium dodecyl sulfate-polyacrylamide gel electrophoresis (10% separation gel), and then transferred to a PVDF membrane. The membrane was blocked with a Tris-buffered saline buffer containing 5% BSA and 0.5% Tween 20 for 1 hour. And then incubated the membrane at 4°C overnight with the primary antibody. Following this, the membrane was incubated with the HRP-conjugated secondary antibody at room temperature for 1 hour. Protein bands were detected by ECL western blotting substrate (Cat No. WBLUF0500, EMD Millipore Corp, USA). Protein quantitative analysis was conducted using Image Lab 3.0 and ImageJ software. Protein levels were normalized to β-actin or GAPDH levels and expressed as a percentage of the untreated control.

For co-immunoprecipitation studies, cells or tissues were lysed using RIPA buffer. A small portion of the lysate was taken out as input. The remaining lysate was incubated with the targeting primary antibody overnight. The next day, magnetic beads (Cat No. LSKMAGAG02, EMD Millipore Corp, USA) were added to the lysate and incubated for 4 hours. Subsequently, the magnetic beads were collected using a magnetic frame, and the supernatant was discarded. The magnetic beads were then washed with RIPA buffer, followed by the addition of buffer containing SDS. The binding proteins on the magnetic beads were eluted in a metal bath at 95°C for 6 minutes. The eluted samples were

stored for subsequent western blot experiments.

LC-MS/MS Analysis

USP7 antibody was added to the myocardial tissue protein lysate of HFpEF mice for IP experiment, while an IgG antibody was used as the negative control. LC-MS/MS analysis was conducted by OE Bio Co, Ltd (Shanghai, China). Based on the scores and quality of the detected proteins, we conducted a screening to identify substrate proteins that could bind to USP7.

Statistical Analysis

Data were reported as Mean±SEM. For $n \geq 6$ data, the Shapiro-Wilk normality test was conducted to assess the normality of the data. Fisher's exact test was utilized to compare categorical variables. For data with a normal distribution, the unmatched two-tailed Student's t-test was employed to determine whether the difference between the two groups was statistically significant. For multi-group comparison, one-way or two-way ANOVA with Tukey's multiple comparison test or Šidák test was utilized. For datasets with $n < 6$ or non-normal distribution, the non-parametric unpaired Mann-Whitney test was used to assess the statistical significance of the difference between the two groups. A statistically significant difference was obtained at $P < 0.05$. Data were analyzed by GraphPad Prism software (version 9.4.1, CA, USA) and R (Version 4.2.3).

Supplemental Figures

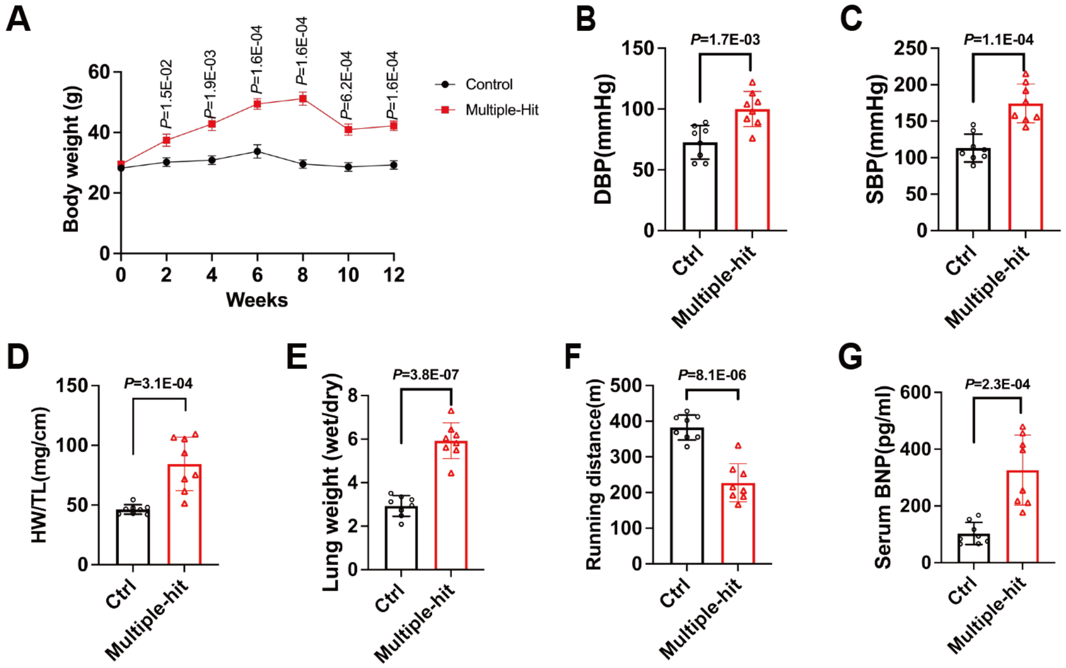

Figure S1. "Multiple-hit" strategy recapitulates obesity, hypertension and heart failure in mice. **A,** Body weight of the mice under normal diet and "Multiple-hit" strategy group. **B** and **C**, diastolic blood pressure (DBP) and Systolic blood pressure (SBP) of two group. **D**, Heart weight (HW) normalized to tibia length (TL). **E**, Ratio between wet and dry lung weight. **F**, Running distance during exercise exhaustion test. **G**, The serum levels of NT-proBNP (N-terminal pro-B-type natriuretic peptide) in two groups. **A-G**, Student t test; number of comparisons = 16.

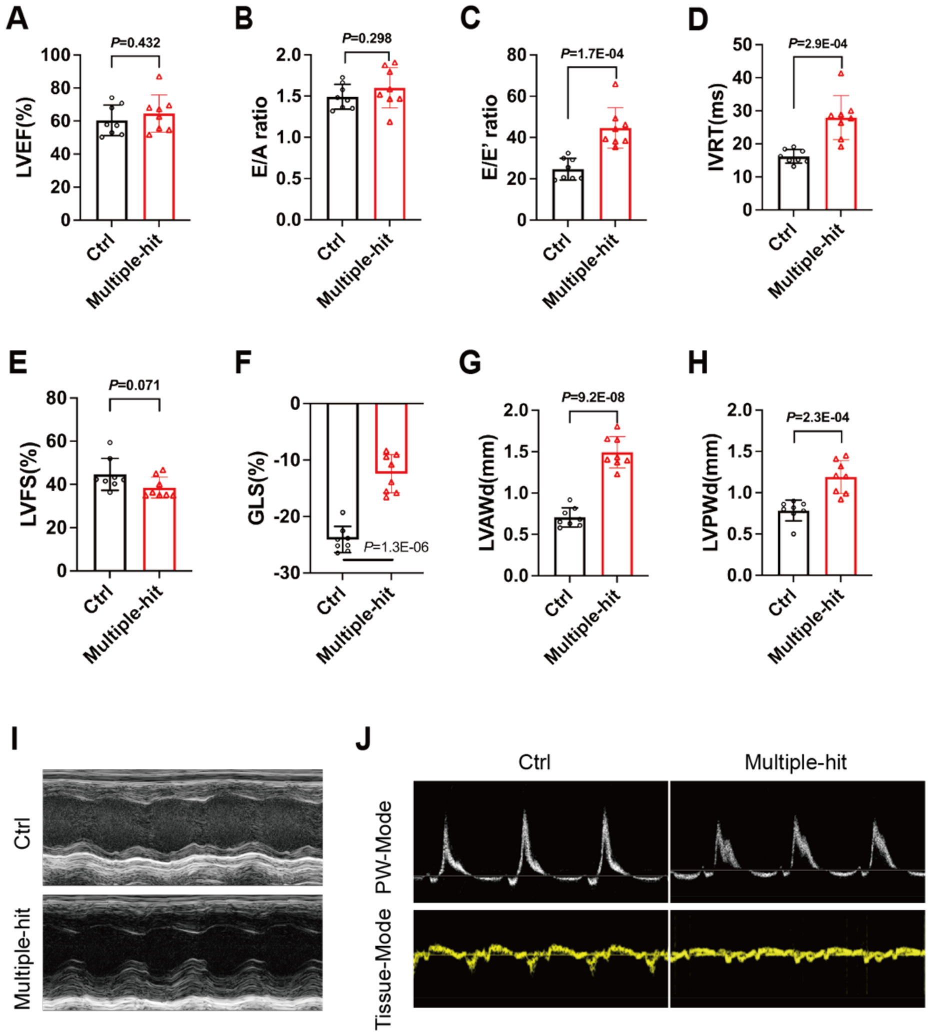

Figure S2. "Multiple-hit" strategy causes diastolic dysfunction but preserves ejection fraction.

A, Percentage of left ventricular ejection fraction (LVEF) under normal diet and "Multiple-hit" strategy group;
B, Ratio between mitral E wave and A wave (E/A);
C, Ratio between mitral E wave and E′ wave (E/E′);
D, Isovolumic relaxation time (IVRT);
E, Percentage of left ventricular fraction shortening (LVFS);
F, Percentage of global longitudinal strain (GLS);
G-H, left ventricular anterior wall thickness in diastole (LVAWd) and left ventricular posterior wall thickness in diastole (LVPWd);
I, Representative left ventricular M-mode echocardiographic tracings in long-axis view;
J, Representative pulsed-wave Doppler tracings;
A-H, Student t test; number of comparisons = 16.

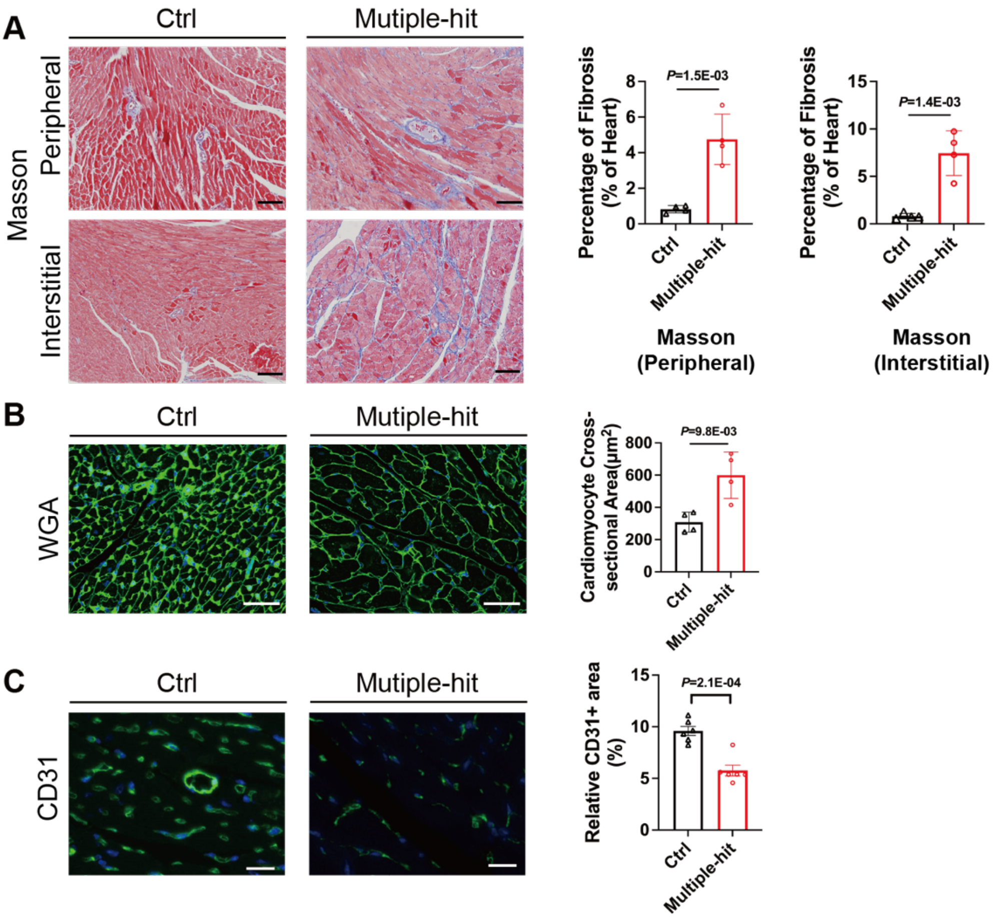

Figure S3. "Multiple-hit" strategy causes myocardial fibrosis, cardiac hypertrophy and microvascular rarefaction.

A, Representative images of Masson and quantification of fibrosis by assessing the Masson areas in sections of hearts. Scale bar, 100 μm, n = 4;

B, Representative images of wheat germ agglutinin (WGA) staining and quantification of cardiomyocyte cross-sectional area based on WGA staining. n = 4, with 150 to 300 myocytes analyzed per image. Scale bars, 50 μm, n = 4;

C, Representative images and quantification of of capillary density ($CD31^+$). Scale bars, 20 μm, n = 4;

A-C, Student t test; number of comparisons = 8.

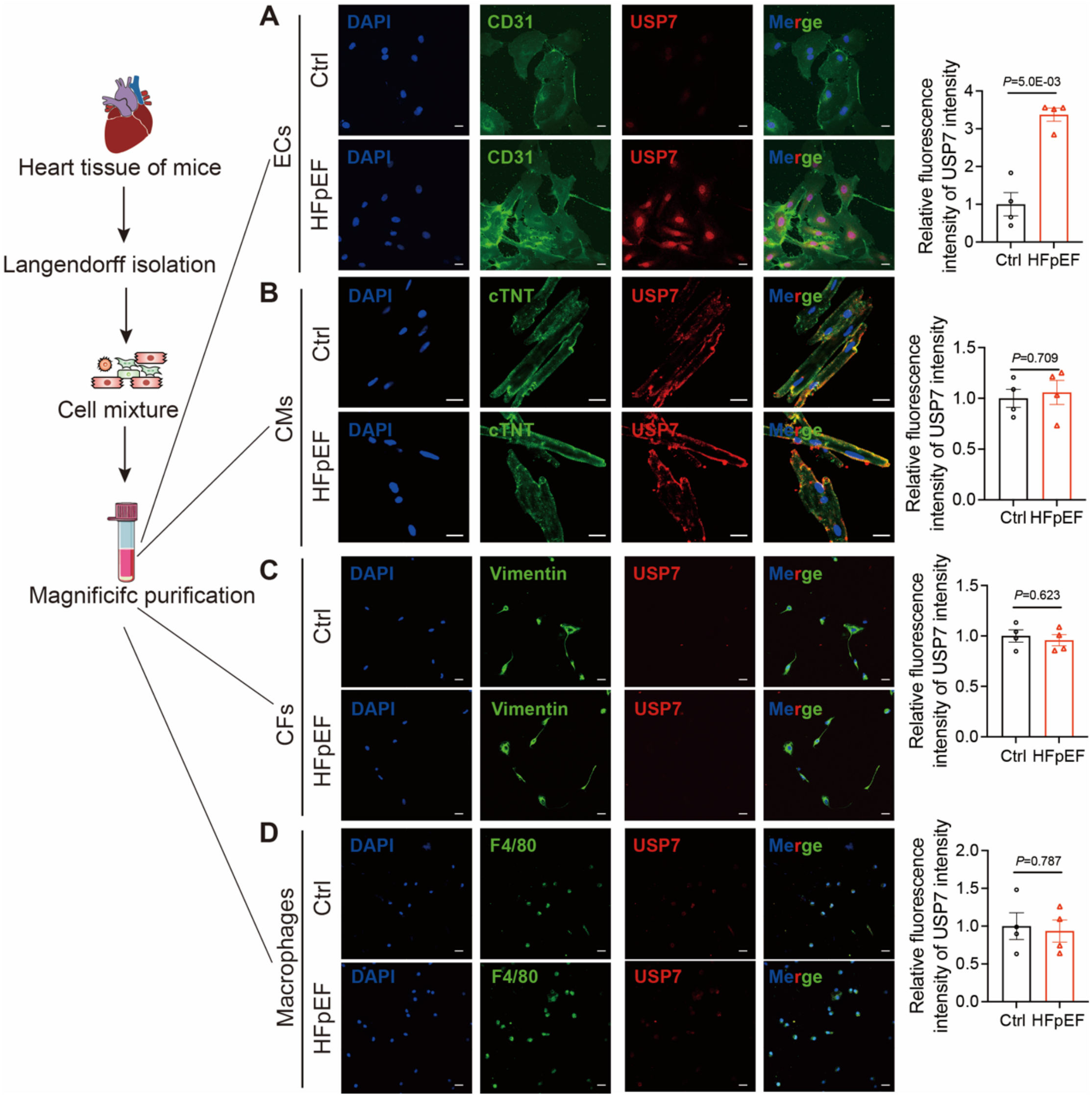

Figure S4. The upregulation of USP7 was primarily localized in the endothelial cells. Schematic outline of the isolation of cellular fractions from myocardial tissue in HFpEF mice. Immunostaining for ECs (CD31+), cardiomyocytes (cTNT+), cardiac fibroblasts (Vimentin+) and macrophages (F4/80). ECs, endothelial cells; CMs, cardiomyocytes; CFs, cardiac fibroblasts.

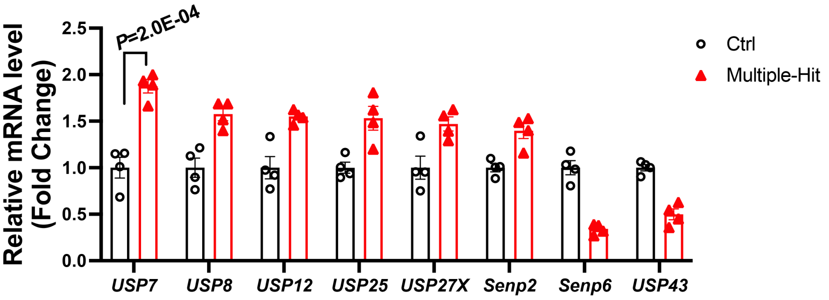

Figure S5. Real-time qPCR analysis of the mRNA expression of partial DUBs in heart tissues from control and Multiple-hit mice. the mRNA level from each group was normalized to 1 value from the control/nonhypertrophy group, which was set to 1. n = 4.

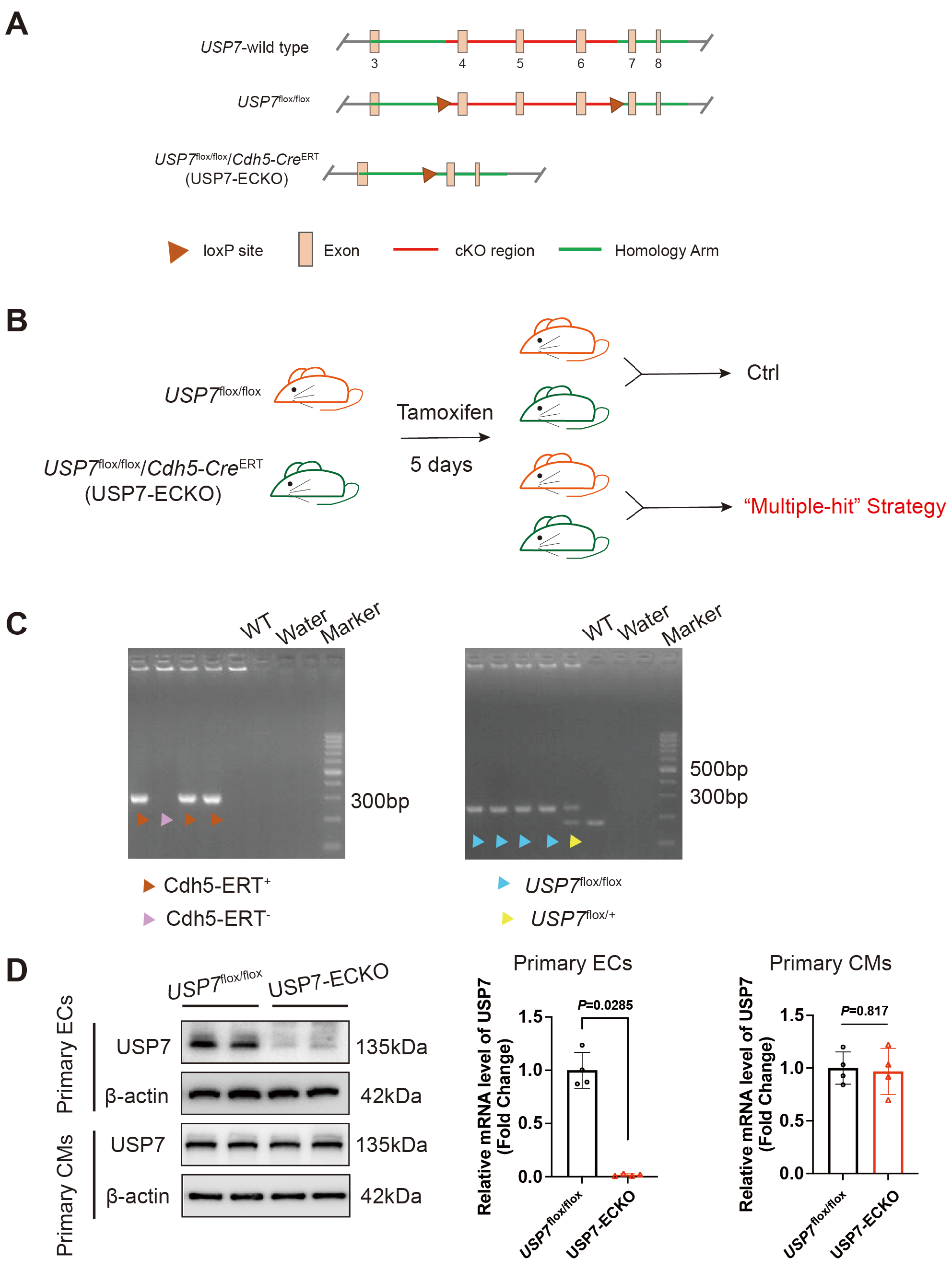

Figure S6. Gene identification and validation of EC-specific USP7 knockout mice.
A, Schematic diagram for generation of USP7-ECKO mice. **B**, Schematic diagram for grouping strategy. **C**, Representative images of polymerase chain reaction of DNA isolated from tails in *USP7*$^{flox/flox}$ and *USP7*$^{flox/flox}$/Cdh5-Cre^{ERT} mice. **C-D**, Representative western blot and RT-qPCR analysis of USP7 protein levels in cardiac endothelial cells (ECs) and cardiomyocytes (CMs) isolated from *USP7*$^{flox/flox}$ and USP7-ECKO mice.

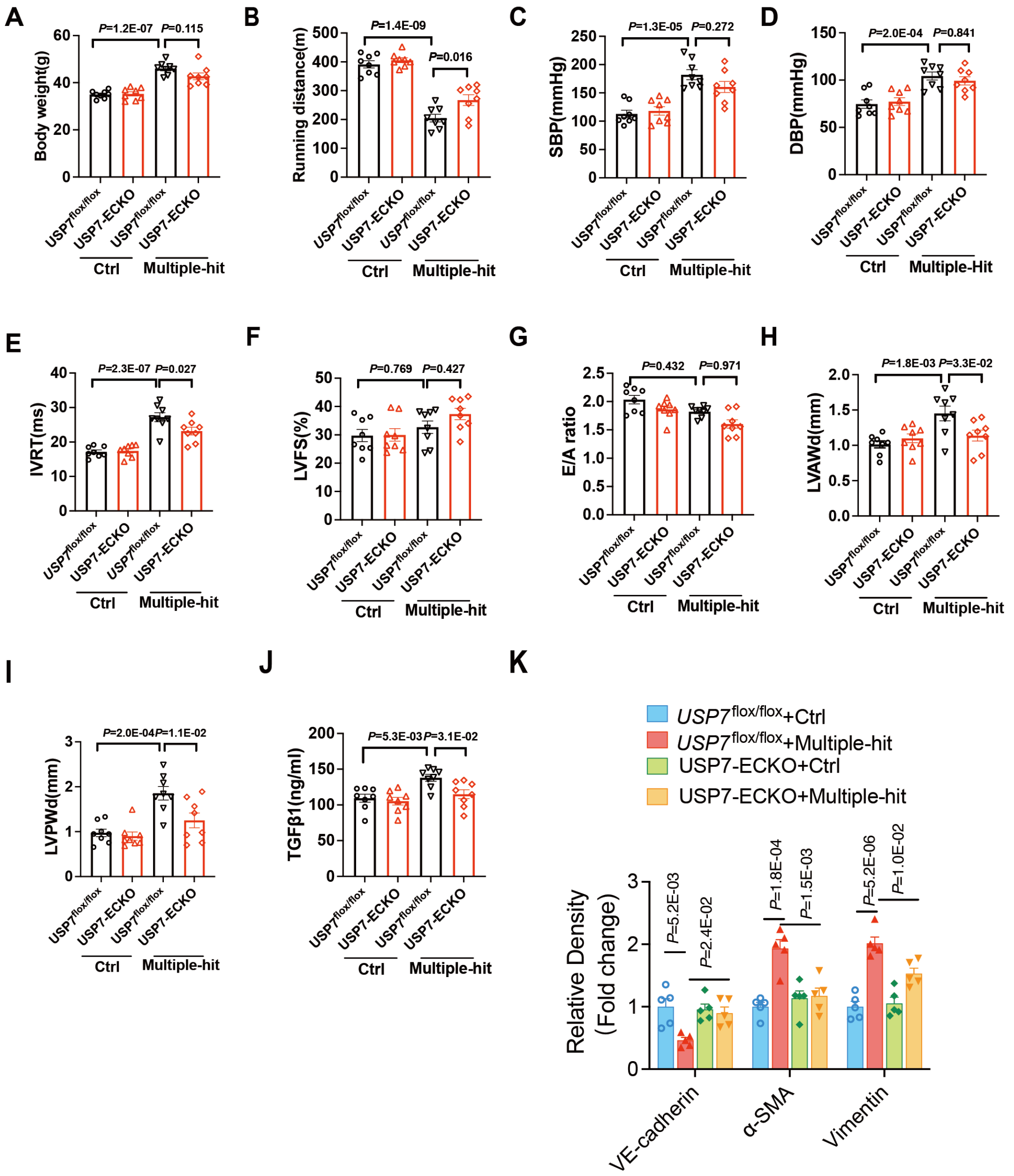

Figure S7. EC-specific knockout of USP7 not alleviates body weight, blood pressure, E/A ratio and left ventricular fraction shortening (LVFS).

A, Body weight of *USP7*$^{\text{flox/flox}}$ and USP7-ECKO mice under "Multiple-hit" or not. **B**, Running distance during exercise exhaustion test. **C and D,** Systolic blood pressure (SBP) and diastolic blood pressure (DBP). **E,** Isovolumic relaxation time (IVRT). **F**, Left ventricular fraction shortening (LVFS). **G**, Ratio between mitral E wave and A wave (E/A). **H**, Left ventricular anterior wall thickness in diastole (LVAWd). **I,** Left ventricular posterior wall thickness in diastole (LVPWd). **J**, The serum levels of TGFβ1 in four groups. **K**, Densitometric quantification of western blot analysis for endothelial cell marker (VE-cadherin), mesenchymal marker (α-SMA, Vimentin) from the heart tissues of four groups.

A-K, 1-way ANOVA followed by Tukey post-hoc tests; Data are shown as mean ± SEM and adjusted P values were provided in case of multiple groups.

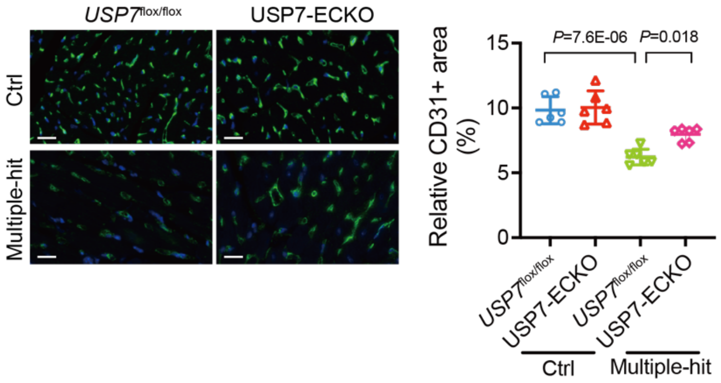

Figure S8. EC-specific knockout of USP7 alleviates microvascular rarefaction of HFpEF mice. **A and B**, Representative images and quantification of of capillary density ($CD31^+$). Scale bars, 20 µm. **B**, 1-way ANOVA followed by Tukey post-hoc tests; Data are shown as mean±SEM and adjusted P values were provided in case of multiple groups.

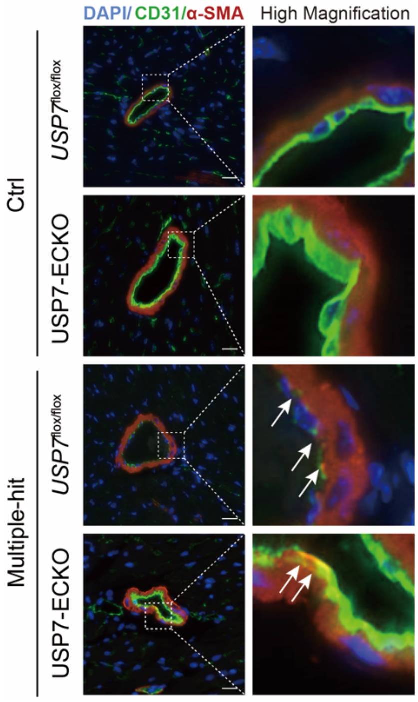

Figure S9. EC-specific knockout of USP7 alleviates the EndMT process in big vascular in hearts.

Representative immunofluorescent staining images of big vascular endothelial cell CD31 (green) and fibrosis marker α-SMA (red) in the heart tissues of control and HFpEF mice (n = 4 per group). Scale bar, 20 µm.

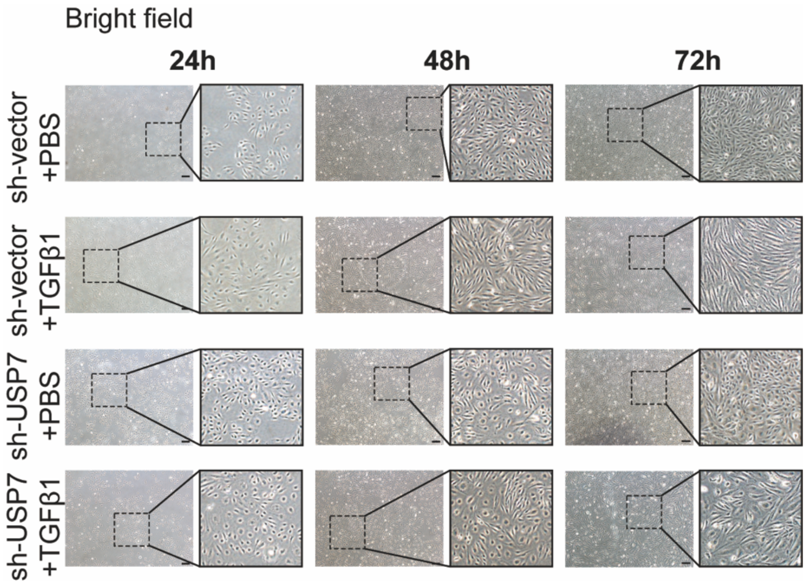

Figure S10. Knocking down USP7 alleviates endothelial EndMT in vitro.

Representative bright field images in transfected CMECs (sh-vector or sh-USP7) were either untreated or treated with TGFβ1 for 24, 48 and 72 hours. Scale bars, 200 μm.

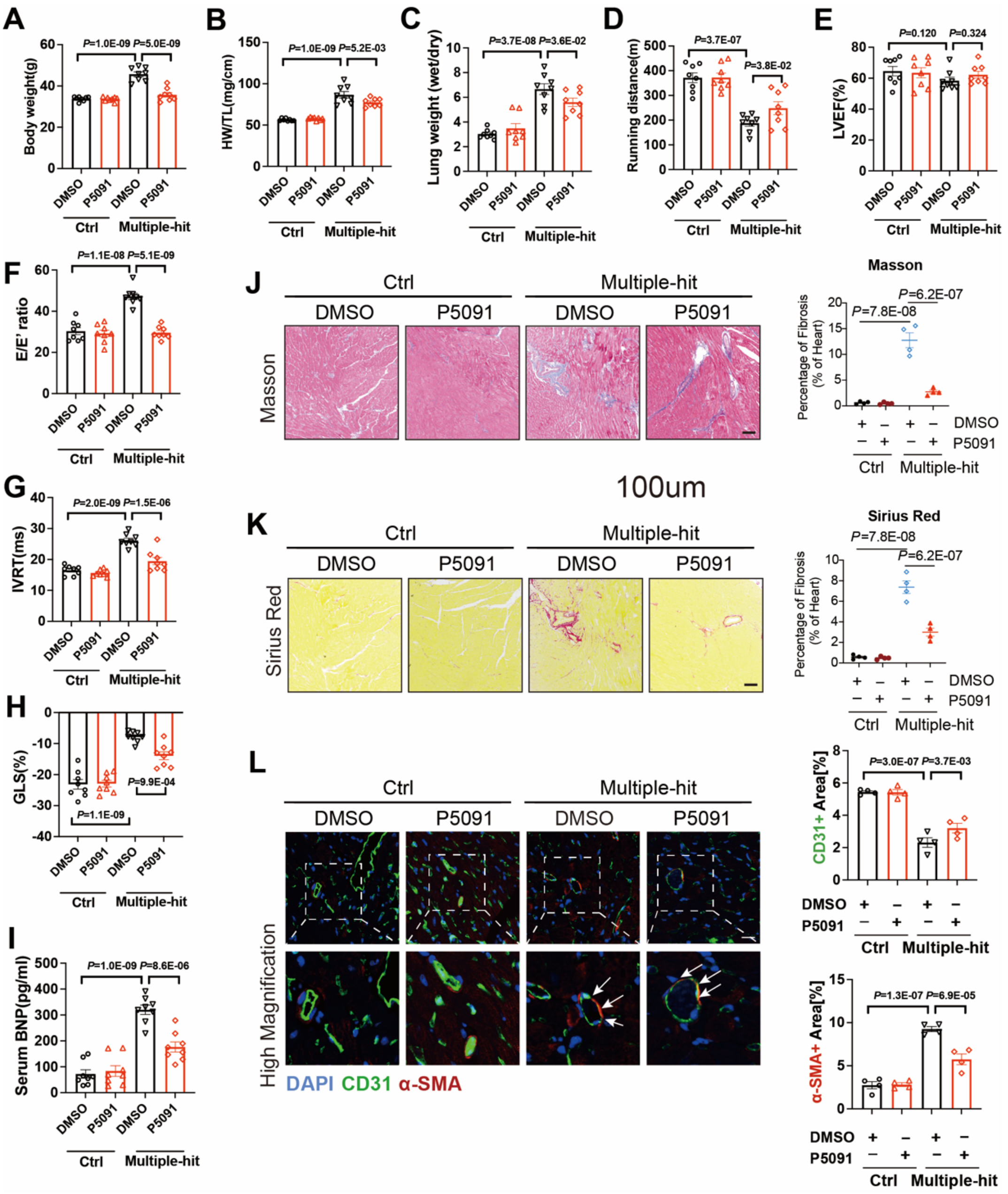

Figure S11. USP7 inhibitor P5091 alleviates cardiac fibrosis by mitigating EndMT, thereby ameliorating the HFpEF phenotypes.

Four group of WT mice were subjected to normal diet and “Multiple-hit” strategy and with the injection of DMSO or P5091.

A, Body weight of 4 groups of mice. **B,** Heart weight (HW) normalized to tibia length (TL). **C**, Ratio between wet and dry lung weight. **D**, Running distance during exercise exhaustion test. **E**, Percentage of left ventricular ejection fraction (LVEF). **F**, Ratio between mitral E wave and E′ wave (E/E′). **G**, Isovolumic relaxation time (IVRT). **H**, Percentage of global longitudinal strain (GLS). **I**, The serum levels of BNP (B-type natriuretic peptide) in four groups. **J and K**, Masson (**J**) and Sirius Red (**K**) staining and quantification in sections of hearts. (Scale bar, 100 μm). **L**, Representative immunofluorescent staining images and quantification of microvascular endothelial cell CD31 (green) and fibrosis marker α-SMA (red) in the heart tissues of control and HFpEF mice (n = 4 per group). Scale bar, 20 μm.

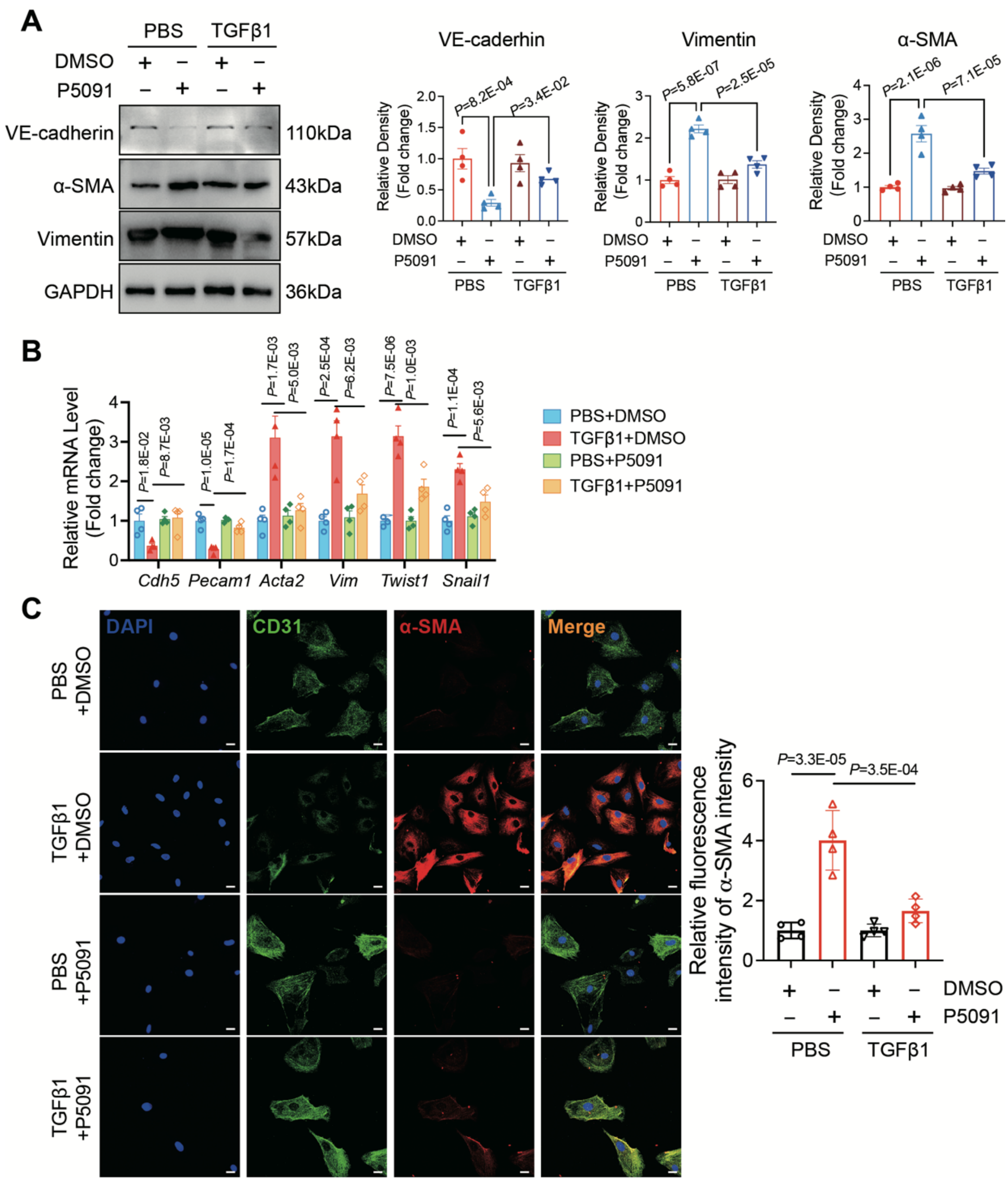

Figure S12. USP7 inhibitor P5091 alleviates endothelial EndMT in vitro.

A, Representative western blotting analysis and densitometric quantification of endothelial cell marker (VE-cadherin) and mesenchymal marker (α-SMA, Vimentin) in primary cardiac microvascular endothelial cells (CMECs) treated with P5091(1 μM, 24h) or DMSO under TGFβ1 stimulation (10 ng/ml, 72h) or PBS control. n = 4. **B**, Expression analysis by RT-qPCR of endothelial marker (Cdh5 and Pecam1), mesenchymal marker (Acta2 and Vim) and transcription factors of EndMT (Twist1 and Snail1) in primary cardiac microvascular endothelial cells (CMECs) treated with P5091(1 μM, 24h) or DMSO under TGFβ1 stimulation under TGFβ1 stimulation (10 ng/ml, 72h) or PBS control. n = 4. **C**, Immunofluorescence staining of CD31 (green) and α-SMA (red) (H) treated with P5091(1 μM, 24h) or DMSO under TGFβ1 stimulation (10 ng/ml, 72h) or PBS control. Scale bar, 20 μm.

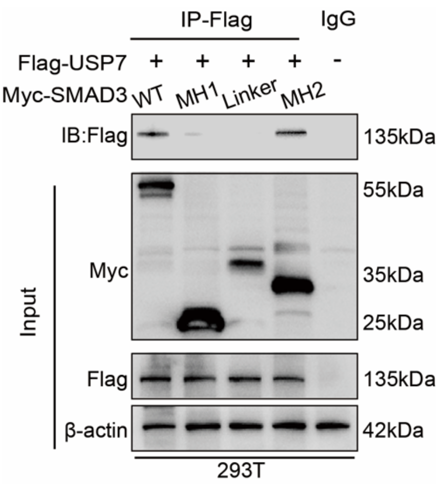

Figure S13. SMAD3 interacted with MH2 domain with USP7.

Coimmunoprecipitation of WT-SMAD3, Mut-SMAD3, and USP7 in 293T cells co-transfected with overexpression plasmids of Myc-SMAD3 (WT), Myc-SMAD3 (MH1), Myc-SMAD3 (Linker), Myc-SMAD3 (MH2) and Flag-USP7. Exogenous normal or mutated SMAD3 was immunoprecipitated by anti-Myc antibody.

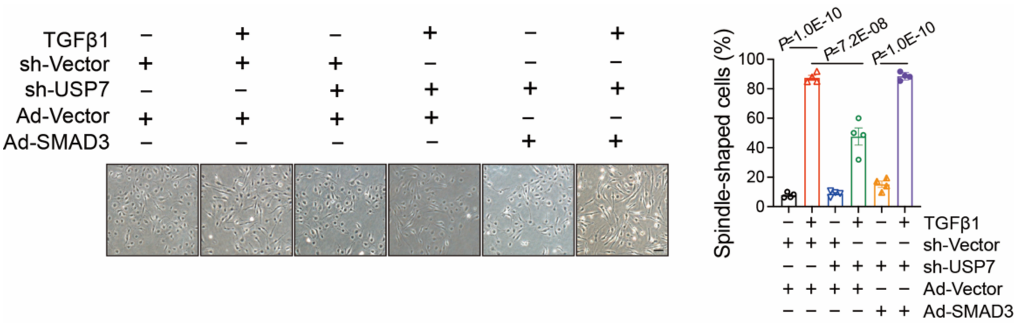

Figure S14. The improvement in the EndMT process resulting from the knockdown of USP7 was partially attenuated by overexpressing SMAD3.

Bright field image in primary cardiac microvascular endothelial cells (CMECs) cotransfected with short hairpin RNA lentiviral particles targeting USP7 (shUSP7)/control adenovirus (sh-Vector) and control adenovirus(ad-Vector)/recombinant SMAD3 adenovirus (Ad-SMAD3) under TGFβ1 stimulation (10 ng/ml, 72h) or PBS control. Quantification of EndMT of CMECs by assessing the percentage of spindle-shaped cells (C). n = 4. Scale bar, 20 μm.

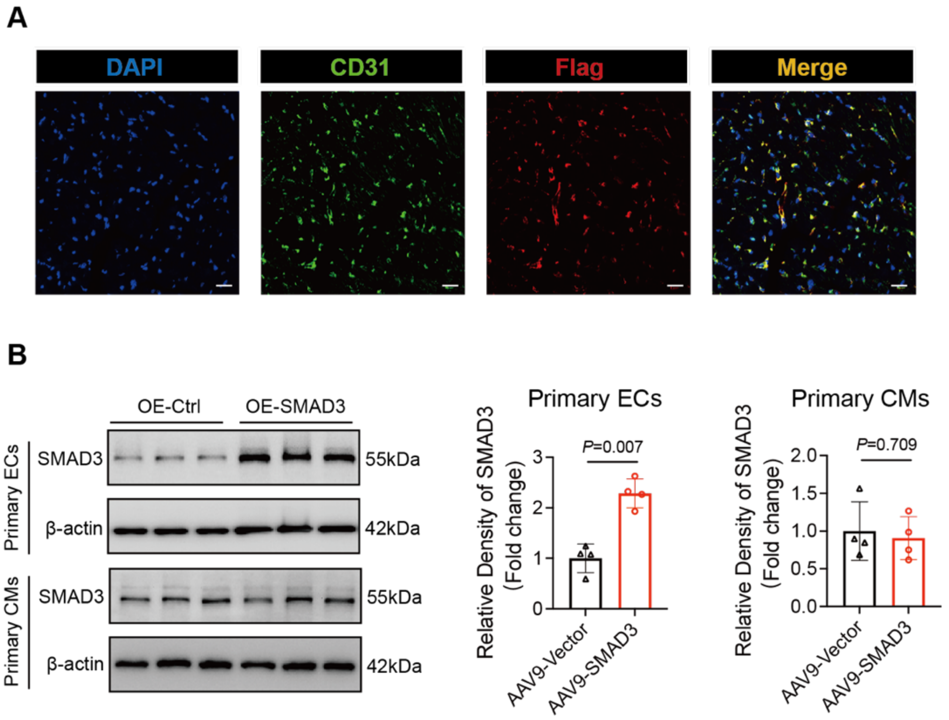

Figure S15. SMAD3 was highly expressed in cardiac ECs of HFpEF mice by AAV9-ENT via cardiac injection in situ.

A, Immunofluorescence staining of CD31 (green) and Flag (red) in AAV9-ENT-ICAM2-infected hearts. Scale bar = 20 μm. B Immunoblotting was performed to quantify SMAD3 expression in ECs and cardiomyocytes isolated from AAV9-ENT-ICAM2-control (OE-Ctrl) or AAV9-ENT-ICAM2-SMAD3 (OE-SMAD3) infected hearts; n = 3 mice per group.

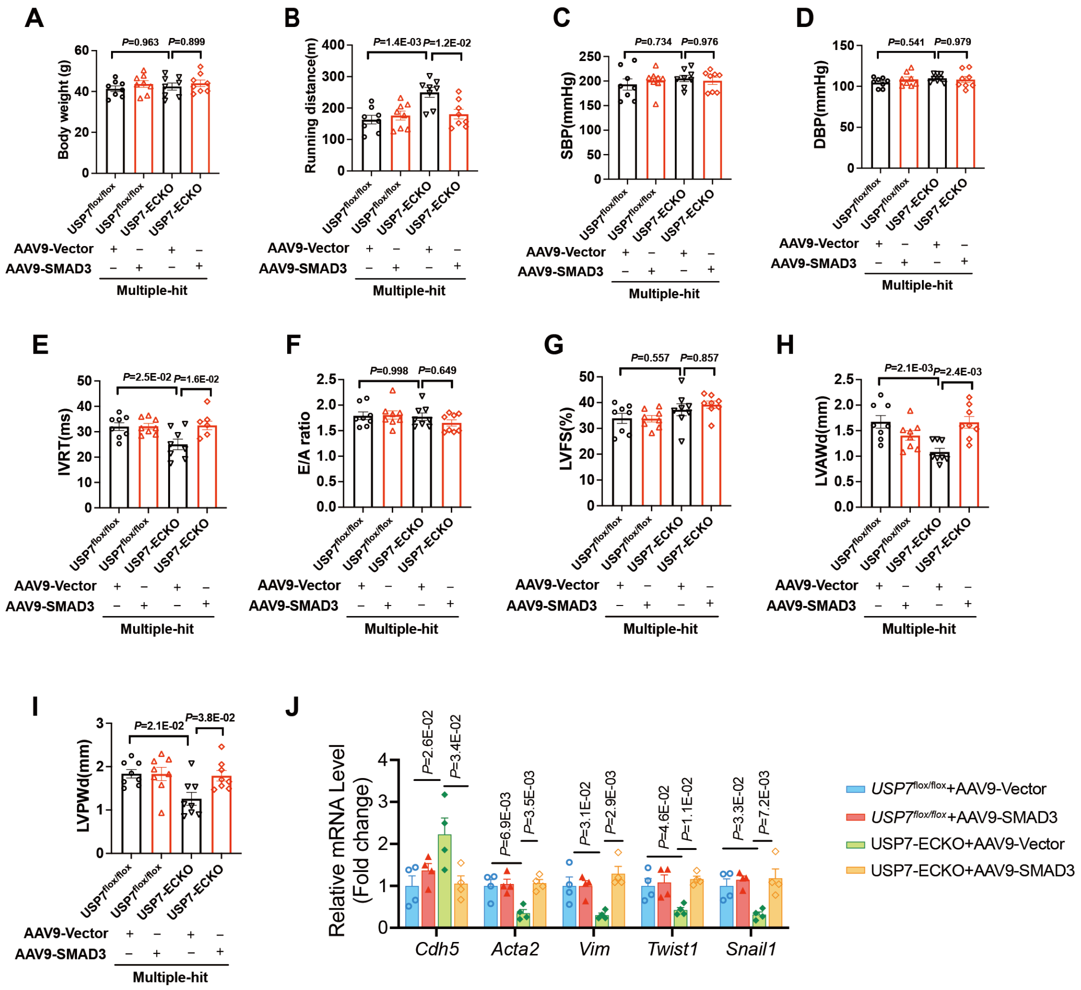

Figure S16. Overexpression of SMAD3 in ECs not effect on body weight, blood pressure and left ventricular fraction shortening (LVFS)

A, Body weight of *USP7*$^{flox/flox}$ and USP7-ECKO mice under "Multiple-hit" in different cardiac injection strategy. **B,** Running distance during exercise exhaustion test. **C and D,** Systolic blood pressure (SBP) and diastolic blood pressure (DBP). **E,** Isovolumic relaxation time (IVRT). **F,** Ratio between mitral E wave and A wave (E/A). **G,** left ventricular fraction shortening (LVFS). **H,** Left ventricular anterior wall thickness in diastole (LVAWd**). I,** Left ventricular posterior wall thickness in diastole (LVPWd). **J,** Real-time qPCR analysis of endothelial cell marker (Cdh5), mesenchymal marker (Acta2, Vim) and transcription factors of EndMT (Twist1, Snail1) in heart tissues. n = 4. **A-J**, 1-way ANOVA followed by Tukey post-hoc tests; Data are shown as mean ± SEM and adjusted P values were provided in case of multiple groups.

Supplemental Tables

Table S1 Part of DUBs in expression from RNA-sequence data between Ctrl and Multiple-hit group

Rowname	logFC	AveExpr	t	P.Value	adj.P.Val	B
Usp7	0.82356658	6.54553457	8.78541296	8.96E-05	0.00386744	1.44342774
Usp8	0.76852895	7.42062399	6.52132778	5.00E-04	0.00906404	-0.4966435
Usp12	0.94090835	7.41129351	6.00841218	7.87E-04	0.01159774	-1.0088766
Usp25	0.83636007	7.78730398	5.3618996	0.00145708	0.01657143	-1.7007822
Senp6	-0.8265075	5.5555181	-4.9587006	0.00219658	0.02138608	-2.1605023
Usp27x	1.42046961	3.03180205	4.50334318	0.00358636	0.02895158	-2.7073297
Senp2	0.69368979	7.09754466	4.49423066	0.00362281	0.0291276	-2.718579
Usp43	-1.2655998	5.94270872	-3.9594176	0.00670123	0.04365002	-3.4000027

Table S2 Conventional and Doppler echocardiographic indices of LV systolic and diastolic function in *USP7*$^{flox/flox}$ and USP7-ECKO mice

Parameter	*USP7*$^{flox/flox}$+ND n=8	USP7-ECKO+ND n=8	*USP7*$^{flox/flox}$ +Multiple hit n=8	USP7-ECKO+Multiple hit n=8
HR (bpm)	457.09±9.75	490.47±11.37	481.97±12.58	487.91±13.99
LVIDs (mm)	2.13±0.21	1.99±0.08	2.19±0.20	1.90±0.15
LVIDd (mm)	3.87±0.15	3.86±0.12	3.82±0.09	3.88±0.16
LVESV (μL)	32.88±6.05	33.86±5.37	20.13±2.93	18.49±3.30
LVEDV (μL)	76.18±9.10	82.43±22.56	57.25±6.97	62.04±7.68
Stroke volume(μL)	43.31±3.40	48.57±2.84	37.12±4.94	43.55±5.15
EF (%)	59.61±3.89	61.11±3.32	65.06±3.62	70.64±3.02
FS (%)	29.74±2.15	29.97±2.22	32.68±2.21	37.37±2.00
LVAWd (mm)	1.01±0.05	1.10±0.06	1.45±0.10**	1.14±0.08#
LVAWs (mm)	1.46±0.09	1.65±0.08	2.16±0.13**	1.74±0.12#
LVPWd (mm)	0.97±0.08	0.90±0.09	1.86±0.15**	1.25±0.17#
LVPWs (mm)	1.29±0.10	1.16±0.10	2.22±0.11**	1.63±0.15##
IVCT (ms)	17.14±0.50	17.16±0.45	20.66±1.48	20.17±0.98
IVRT (ms)	17.15±0.54	17.43±0.62	27.21±1.30**	23.17±1.12#
Peak mitral E velocity (mm/s)	724.17±16.41	697.54±14.60	748.87±15.42	724.20±14.13
Peak mitral E' velocity (mm/s)	-25.29±1.27	-24.38±0.65	-19.73±1.24*	-25.48±1.33##
Peak mitral A velocity (mm/s)	358.25±10.97	377.24±13.24	411.60±10.13*	456.71±17.95

MV E/A	2.03±0.08	1.86±0.06	1.82±0.03	1.60±0.07
MV E/E’	-29.17±1.71	-28.72±0.84	-38.77±1.93**	-29.07±1.82##
GLS	-23.34±1.13	-23.35±1.20	-9.45±0.96**	-14.19±1.32#

A, peak Doppler blood inflow velocity across mitral valve during late diastole; E, peak Doppler blood inflow velocity across mitral valve during early diastole; E’, peak tissue Doppler of myocardial relaxation velocity at mitral valve annulus during early diastole; EF, ejection fraction; FS, fractional shortening; GLS, global longitudinal strain as marker of myocardial deformation; HR, heart rate; IVRT, isovolumic relaxation time; LVAWd, left-ventricular end-diastolic anterior wall thickness; LVAWs, left-ventricular end-systolic anterior wall thickness; LVEDV, left ventricular end-diastolic volume; LVESV, left ventricular end-systolic volume; LVIDd, left ventricular end-diastolic diameter; LVIDs, left ventricular end-systolic diameter; Results are presented as mean ± SE. one-way ANOVA plus Sidak’s multiple-comparisons test. *P<0.05 and **P<0.01 vs. *USP7*$^{flox/flox}$+ND; #P<0.05 and ##P<0.01 vs. USP7-ECKO+Multiple-hit.

Table S3 Conventional and Doppler echocardiographic indices of LV systolic and diastolic function in the *USP7*$^{flox/flox}$ and USP7-ECKO HFpEF mice under *AAV9-Vector* or *AAV9-SMAD3* injection

**Parameter**	***USP7*$^{flox/flox}$ *AAV9-Vector* n=8**	***USP7*$^{flox/flox}$ *AAV9-SMAD3* n=8**	**USP7-ECKO *AAV9-Vector* n=8**	**USP7-ECKO *AAV9-SMAD3* n=8**
HR (bpm)	471.00±18.61	487.82±17.68	476.17±9.54	466.83±13.70
LVIDs (mm)	2.45±0.18	2.05±0.11	1.88±0.10*	1.97±0.11
LVIDd (mm)	4.27±0.24	3.94±0.10	3.79±0.11	4.04±0.10
LVESV (μL)	28.24±8.08	18.30±5.63	11.07±1.61	7.45±1.58
LVEDV (μL)	71.18±17.56	50.33±10.54	38.31±3.88	30.57±2.43
Stroke volume(μL)	42.94±9.76	32.03±5.29	27.23±3.26	23.13±1.49
EF (%)	62.50±2.34	68.57±4.30	70.44±4.24	76.44±3.31
FS (%)	33.89±1.96	33.76±1.26	37.22±2.33	39.23±1.37
LVAWd (mm)	1.67±0.12	1.40±0.09	1.08±0.07**	1.66±0.12##
LVAWs (mm)	2.34±0.15	2.10±0.12	1.70±0.07**	2.35±0.13##
LVPWd (mm)	1.84±0.10	1.83±0.15	1.26±0.15*	1.79±0.12#
LVPWs (mm)	2.15±0.16	2.16±0.11	1.57±0.15*	2.14±0.16#
IVCT (ms)	24.83±2.07	19.69±1.16	22.83±0.95	20.45±1.04
IVRT (ms)	32.01±1.54	32.16±1.10	25.01±2.05*	32.47±1.69#
Peak mitral E velocity (mm/s)	732.03±12.24	704.36±16.62	695.86±17.17	692.49±9.66
Peak mitral E' velocity (mm/s)	-17.27±0.99	-17.18±1.04	-21.66±1.02*	-17.29±1.06#
Peak mitral A velocity (mm/s)	412.91±14.77	393.27±13.09	396.87±16.80	423.12±13.96
MV E/A	1.79±0.08	1.81±0.08	1.77±0.07	1.65±0.06
MV E/E’	-43.26±2.29	-41.79±2.07	-32.60±1.59**	-40.94±2.12#
GLS	-11.27±1.22	-10.98±1.19	-15.95±0.83*	-11.61±1.17#

A, peak Doppler blood inflow velocity across mitral valve during late diastole; E, peak Doppler blood inflow velocity across mitral valve during early diastole; E’, peak tissue Doppler of myocardial relaxation velocity at

mitral valve annulus during early diastole; EF, ejection fraction; FS, fractional shortening; GLS, global longitudinal strain as marker of myocardial deformation; HR, heart rate; IVRT, isovolumic relaxation time; LVAWd, left-ventricular end-diastolic anterior wall thickness; LVAWs, left-ventricular end-systolic anterior wall thickness; LVEDV, left ventricular end-diastolic volume; LVESV, left ventricular end-systolic volume; LVIDd, left ventricular end-diastolic diameter; LVIDs, left ventricular end-systolic diameter; Results are presented as mean ± SE. one-way ANOVA plus Sidak's multiple-comparisons test. *P < 0.05 and **P < 0.01 vs. *USP7*$^{flox/flox}$ *AAV9-Vector*; #P < 0.05 and ##P < 0.01 vs. USP7-ECKO *AAV9-SMAD3*.

Table S4 Primers used for the RT-qPCR assays in this study.

**Gene name**	**Forward primers (5'- 3')**	**Reverse primers (5'- 3')**
Usp7(mouse)	CCACAAGGAAAACGACTGGG	GTAACACGTTGCTCCCTGATT
Usp8(mouse)	CTCCGGAGTCTGAAAGATGC	CATTGCGCAGGACAGTTTTA
Usp12(mouse)	CCGGGATCCGCCACCATGGAAATCCTAATGACAG	CCGAAGCTTTTTCTATCAGTCTCGGGAC
Usp25(mouse)	GTGTTACCGACGATCCGTGTC	GTTCATGCAGATATAGAGGCCAC
Senp6(mouse)	ATGCAGACAAAGATGGGGCA	CAGTCTTGCTCCGCCTTACA
Usp27x(mouse)	CCCACGGAGAAGAAGGATCG	CTGCTAGATGACGAGCGTG
Senp2(mouse)	TCTGGTGCTGAGTGAATGTGA	GTTGAATGGGAGTGACTGTGG
Usp43(mouse)	GCAGAGGAGGTGATCTTGGTTGAAC	CTTGTGCTCGCCGACTCTGTTC
Cdh5(rattus)	TGAAATGGTGCTTCGGTGCTCTG	GACTGGTCACAATGCTGGCTCTG
Pecam1(rattus)	TGAAATGGTGCTTCGGTGCTCTG	GACTGGTCACAATGCTGGCTCTG
acta2(rattus)	CAACTGGTATTGTGCTGGACTCTGG	TCACGGACGATCTCACGCTCAG
Vim(rattus)	TGACCGCTTCGCCAACTACATC	AACTCCCTCATCTCCTCCTCGTAG
twist1(rattus)	CGACGACAGCCTGAGCAACAG	GCCGACTGCTGCGTCTCTTG
Snail1(rattus)	TCACCTTCCAGCAGCCCTACG	CACCAGGAGAGAGTCCCAGATGAG

Smad3(rattus)	AACACTAACTTCCCCGCTGG	TGTGGTTCATCTGGTGGTCG
β-actin(rattus)	GAGGCCCCTCTGAACCCTAAG	ATGCCAGTGGTACGACCAGA

Table S5. List of the sequences of shRNAs

**Gene Symbol**	**No.**	**Species**	**Sequence**
shUSP7	#1	*Rattus*	CCCTGGATTTGTGGTCACATT
	#2	*Rattus*	CCTGCAATGTTAGATAATGAA
	#3	*Rattus*	GCAACTTATGAGGTTCATGTA
Negative control	NA	*Rattus*	TTCTCCGAACGTGTCACGTAA

Supplemental Reference

1. Luo W, Zou X, Wang Y, Dong Z, Weng X, Pei Z, et al. Critical Role of the cGAS-STING Pathway in Doxorubicin-Induced Cardiotoxicity. Circ Res. 2023; 132: e223-e42.
2. Wu X, Liu H, Brooks A, Xu S, Luo J, Steiner R, et al. SIRT6 Mitigates Heart Failure With Preserved Ejection Fraction in Diabetes. Circ Res. 2022; 131: 926-43.
3. Li F, Wang J, Song Y, Shen D, Zhao Y, Li C, et al. Qiliqiangxin alleviates Ang II-induced CMECs apoptosis by downregulating autophagy via the ErbB2-AKT-FoxO3a axis. Life Sci. 2021; 273: 119239.
4. Ackers-Johnson M, Li PY, Holmes AP, O'Brien SM, Pavlovic D, Foo RS. A Simplified, Langendorff-Free Method for Concomitant Isolation of Viable Cardiac Myocytes and Nonmyocytes From the Adult Mouse Heart. Circ Res. 2016; 119: 909-20.
